# IGF2BP2 Drives Thyroid Cancer Dedifferentiation Through m6A-Dependent STAT1 mRNA Destabilization

**DOI:** 10.7150/ijbs.121503

**Published:** 2026-01-01

**Authors:** Rui Chen, Yi-xun Li, Wei-lin Lu, Ke-fei Wu, Yu-xin Wang, Zi-wen Wang, Yi-han Li, Hai-yan Yang, Xu Zhang, Liang Shi, Dong Zhou, Ying Wang, Qiang Ding

**Affiliations:** 1 Department of Breast Surgery, the First Affiliated Hospital with Nanjing Medical University, 300 Guangzhou Road, 210029, Nanjing, People's Republic of China.; 2 Department of Orthopedics, the Affiliated Changzhou Second People's Hospital of Nanjing Medical University, 468 Yanling Middle Road, Changzhou, People's Republic of China.; 3 Affiliated with Changzhou Children's Hospital of Nantong University, 958 Zhongwu Avenue, 213003, Changzhou, People's Republic of China; 4 Department of Thyroid Surgery, the First Affiliated Hospital with Nanjing Medical University, 300 Guangzhou Road, 210029, Nanjing, People's Republic of China.

**Keywords:** IGF2BP2, thyroid cancer dedifferentiation, stemness, STAT1, m6A

## Abstract

Thyroid cancer is the most common endocrine malignancy globally. While papillary thyroid carcinoma (PTC) typically exhibits favorable prognosis, a subset undergoes dedifferentiation into anaplastic thyroid carcinoma (ATC), an aggressive, treatment-refractory subtype with near-universal lethality. However, the molecular driver of this process remains elusive. In this study, we find that IGF2BP2 is upregulated in ATC and correlates with adverse prognosis. Pseudotime trajectory analysis tracks progressively escalating IGF2BP2 expression throughout dedifferentiation. Functionally, IGF2BP2 promotes proliferation, suppresses thyroid differentiation genes (*TSHR*, *SLC26A4*, *SLC5A5*, *TPO*, *PAX8*, *FOXE1*, and* NKX2.1*), and enhances cancer stemness. Mechanistically, integrated multi-omics analysis (RNA-seq, RIP-seq, and MeRIP-seq) reveals that IGF2BP2 binds m6A-modified *STAT1* mRNA, accelerating its decay. STAT1 directly activates transcription of thyroid differentiation genes. Rescue experiments confirms that STAT1 mediates IGF2BP2-driven dedifferentiation. The IGF2BP2-m6A-STAT1 complex is a master regulator of thyroid cancer dedifferentiation, establishing a novel therapeutic target for redifferentiation therapy in advanced thyroid cancer.

## Introduction

Thyroid cancer (THCA), the most prevalent endocrine malignancy globally^1^, is dominated by papillary thyroid carcinoma (PTC) and typically carries a favorable prognosis^2^. However, accumulating evidence supports a dedifferentiation continuum model, wherein a subset of PTCs progressively evolves into poorly differentiated thyroid carcinoma (PDTC) and ultimately, the highly aggressive anaplastic thyroid carcinoma (ATC)^3^-^5^. This dedifferentiation process, a critical manifestation of tumor plasticity, involves the gradual loss of a differentiated state^6^,^7^, quantifiable by the downregulation of thyroid differentiation score (TDS)^8^ and differentiation markers like *TSHR*^9^,^10^, *SLC26A4*^11^,* SLC5A5*, *TPO*, *PAX8*, *FOXE1*, and *NKX2.1*^12^,^13^. ATC is a rare yet highly lethal subtype characterized by loss of SLC5A5 (NIS) and TSHR expression, rendering it refractory to conventional therapies such as radioactive iodine (RAI) and TSH suppression^4^,^14^,^15^. Therefore, innovative strategies targeting the reversal of dedifferentiation state, or restoration of differentiation in anaplastic cells may circumvent therapeutic barriers, resensitize ATC to cytotoxic agents and ultimately improve survival outcomes.

Unraveling the mechanistic basis of dedifferentiation is paramount for effectively targeting tumor plasticity. Key transcriptional factors (TFs) like CREB3L1^4^, CTCF^16^, CRSP8^17^, and SETMAR^12^ have been identified in thyroid cancer as drivers of dedifferentiation events. Beyond transcriptional control, post-transcriptional regulation, exemplified by the dynamic and reversible N6-methyladenosine (m6A) modification, constitutes a crucial layer governing tumor dedifferentiation and stemness^18^-^20^. This process is orchestrated by 'writers' (e.g., METTL3/METTL14 complex), 'erasers' (e.g., FTO, ALKBH5), and 'readers' (e.g., YTHDF, HNRNPA1, and IGF2BP proteins). While aberrant m6A signaling has been linked to dedifferentiation and stemness in various cancers^21^-^24^, the involvement of this pathway and its key regulators in thyroid cancer dedifferentiation is not well defined and warrant further investigation.

Insulin-like growth factor 2 mRNA-binding protein 2 (IGF2BP2), a conserved oncofetal RNA-binding protein (RBP) and m6A 'reader' 25, recognizes m6A-modified sites on target transcripts to enhance mRNA stability, translation, and subcellular localization. Functioning as an oncogenic driver, IGF2BP2 overexpression is prevalent across multiple cancer types, where it fuels pivotal cancer hallmarks^26^-^30^. Recent work from our group further revealed its upregulation and essential role in cell cycle regulation within triple-negative breast cancer (TNBC)^31^.

Here, we identified IGF2BP2 as a key m6A driver of thyroid cancer dedifferentiation and stemness. Clinical cohort analysis revealed pronounced IGF2BP2 upregulation correlating with dedifferentiation trajectories and adverse patient outcomes. Functionally, *IGF2BP2* overexpression in PTC models accelerated dedifferentiation, while its knockdown in ATC systems restored differentiation markers and attenuated stemness-associated phenotypes. Mechanistically, IGF2BP2 exerts its pro-dedifferentiation effects via m6A-dependent destabilization of *STAT1* mRNA, suppressing STAT1-mediated transactivation of thyroid differentiation-related genes. This IGF2BP2-STAT1 axis established a direct link between epitranscriptomic regulation and tumor plasticity, nominating a novel therapeutic target for redifferentiation strategies.

## Materials and Methods

### Human specimens and bioinformatics analysis

For human specimens, 27 pairs of papillary thyroid carcinoma (PTC) and matched adjacent normal tissue were collected at the First Affiliated Hospital of Nanjing Medical University. PTC accounts for ~84 % of all thyroid malignancies, while poorly differentiated (PDTC) and anaplastic (ATC) carcinomas together represent < 6 % and are rarely available as surgically resected paired samples because of their aggressive clinical course. Consequently, all 27 cases in this consecutive series were histopathologically confirmed PTC. The tissues were embedded in paraffin for immunohistochemistry (IHC) staining. Protein expression was semiquantitatively assessed using the H-score (histochemistry score) method. Briefly, the staining intensity was categorized as 0 (negative), 1+ (weak), 2+ (moderate), or 3+ (strong). The percentage of positively stained cells at each intensity level was estimated and multiplied by the corresponding intensity value. The final H-score was calculated using the formula:

H-score = (0×% negative cells) + (1×% weak cells) + (2×% moderate cells) + (3×% strong cells), resulting in a continuous score ranging from 0 to 300. This study was conducted by the ethical standards of Nanjing Medical University, with approval from the Institutional Ethics Committee of the First Affiliated Hospital of Nanjing Medical University (2020-SR-578). Written informed consent was obtained from all participating patients prior to sample collection.

Bulk RNA sequencing datasets were obtained from the The Cancer Genome Atlas (TCGA) and Gene Expression Omnibus (GEO) database. The raw data (GSE29265, GSE65144, GSE76039, and GSE33630) were integrated and preprocessed using R packages, including normalization and batch effect correction. This yielded a final cohort of 216 samples: 52 anaplastic thyroid carcinomas, 69 papillary thyroid carcinomas, 17 poorly differentiated thyroid carcinomas, and 78 normal thyroid tissues. The processed expression matrix was subsequently employed for three analytical objectives: 1 validation of IGF2BP2 expression patterns across histological subtypes, 2 assessment of thyroid differentiation scores, and 3 correlation analysis between IGF2BP2 and established thyroid differentiation-associated genes. The survival information of THCA patients was obtained from the KM plotter (http://www.kmplot.com) and re-analyzed by GraphPad Prism.

Single-cell RNA sequencing data from 7 papillary thyroid carcinomas (PTC, GSE184362) and 5 anaplastic thyroid carcinomas (ATC, GSE148673) were retrieved from the GEO database. Raw counts matrix underwent quality control and preprocessing in Seurat (v5.1.0) using R (v4.4.1). A two-step stringent quality control procedure was implemented: 1 fixed-parameter filtering retaining cells with nFeature-RNA > 500, nCount-RNA > 1000, percent.mt < 20, and percent.ribo < 60; 2 sample-specific dynamic filtering based on median ± 3 median absolute deviations (MAD) for each quality metric per sample. Potential doublets were then identified and removed using DoubletFinder (v2.0.4). Furthermore, we specifically identified and excluded heat-shock responsive cells and stripped-nuclei based on their distinct gene expression signatures. Batch effects across datasets and samples were corrected using harmony (v1.2.0), followed by linear dimensionality reduction with PCA and non-linear reduction and visualization with tSNE. Cell cluster annotation was guided by marker identification from previous studies^4^ and canonical marker genes for specific subpopulations. To comprehensively identify marker genes for each cell subcluster, we integrated results from both the COSG algorithm (v0.9.0) and Seurat's FindAllMarkers function. Pseudotime trajectories were subsequently reconstructed using Monocle (v2.32.0) to infer developmental processes.

### Cell lines and cell culture

The human thyroid follicular epithelial cell line Nthy-ori3-1, PTC cell lines (TPC1, and BCPAP) and ATC cell lines (CAL62, and C643) were procured from the American Type Culture Collection (ATCC, Manassas, VA, USA). The tumor cell lines were cultured in Dulbecco's modified Eagle's medium (Wisent, China) while Nthy-ori3-1 cell was maintained in Roswell Park Memorial Institute 1640 (Wisent, China), collectively supplemented with 10% fetal bovine serum (Wisent, China) and 1% penicillin-streptomycin (Biosharp, China) and incubated in a humid environment with 5% CO_2_ at 37°C.

### Lentivirus transfection, small interfering RNA, and plasmids

Produced by GenePharma (Shanghai, China), lentiviral vectors carrying short hairpin RNA targeting *IGF2BP2* (sh*IGF2BP2*) and a non-targeting control (shNC) were transfected into TPC1 and CAL62 cells, while CAL62 and C643 were infected with overexpression constructs (*IGF2BP2*) and matched negative control (Vector). Lentiviruses were transduced for 24 h, and stable cell lines were selected using 3 μg/mL puromycin (MCE, USA, HY-B1743A).

For rescue experiments, small interfering RNAs (siRNAs) targeting STAT1 were synthesized by Tsingke Biotechnology Co., Ltd. (Nanjing, China) and the overexpression plasmids were designed and synthesized by Corues Biotchnology (Nanjing, China). For transfection, 3×10⁵ cells were pre-seeded in six-well plates overnight. Lipofectamine 3000 transfection reagent (Invitrogen, USA) was used following manufacturer protocols. The information of lentiviruses, siRNA and plasmids used in this study is listed in Table S1.

### Quantitative RT-PCR (qRT-PCR)

Total RNA was extracted with Trizol reagent (TaKaRa, Japan), followed by cDNA synthesis using HiScript II Q RT SuperMix (Vazyme, China) with 1000 ng RNA. The PCR amplification program was performed on StepOnePlus Real-Time PCR system (Applied Biosystems, USA) using FastStart Universal SYBR Green Master (Roche, Switzerland) according to the manufacturer's instructions. Relative quantification was calculated via 2^-ΔΔCT^ or 2^-ΔCT^ method and normalized based on β-actin. The sequences of specific PCR primers were listed in Table S2.

### Western blot

Cells were lysed in RIPA buffer (Beyotime, China, P0013C) containing 1% PMSF, 1% phosphatase inhibitor, and 0.1% protease inhibitor. Cell lysates were resolved by 10% SDS-PAGE gels and transferred to PVDF membranes (Millipore, USA). After blocked with QuickBlock™ Blocking Buffer (Beyotime, China, P0252) for 15min, membranes were incubated with primary antibodies overnight at 4°C and secondary antibodies (1:5000, Cell Signaling Technology, USA, 7074P2) at room temperature for 2 h. The primary antibodies used were as follows: IGF2BP2 (1:1000, Proteintech, China, 11601-1-AP), FOXE1 (1:1000, UpingBio, China, YP-Ab-01724), PAX8 (1:1000, UpingBio, China, YP-Ab-15794), NKX2.1 (1:1000, UpingBio, China, YP-Ab-15626), TSHR (1:1000, UpingBio, China, YP-Ab-07557), TPO, SLC5A5 (1:1000, UpingBio, China, YP-Ab-17263), SLC26A4 (1:1000, UpingBio, China, YP-Ab-07718), CD133 (1:1000, UpingBio, China, YP-Ab-13976), NANOG (1:1000, UpingBio, China, YP-Ab-15732), SOX2 (1:1000, UpingBio, China, YP-Ab-01022), OCT4 (1:1000, UpingBio, China, YP-Ab-15741), STAT1 (1:1000, Proteintech, China, 66545-1-IG), and GAPDH (1:2000, Proteintech, China, 60004-1-IG). GAPDH was used to normalize protein loading. The antibodies were diluted according to the manufacturer's instructions.

### Cell counting kit-8 (CCK-8) assay

CCK-8 (Vazyme, China) assays were performed according to the manufacturer's instructions. 500-1000 cells were seeded in a 96-well plate. 10% CCK-8 reagent was added at designated timepoints every day, and then the cells were incubated at 37°C for 2 h away from light. Absorbance at 450 nm was measured using a microplate reader (5082 Grodig, Tecan, Austria).

### 5-Ethynyl-2′-Deoxyuridine (EdU) assay

Cell proliferation was assessed using EdU Cell Proliferation Kit (Beyotime, China, C0075). Cells (3 × 10^4^/well) in 24-well plates were incubated with 10 μM EdU per well for 2h. Then, these cells were fixed with 4% paraformaldehyde and stained with 1× click reaction buffer and 1× Hoechst 33342 solution. Finally, the cells were detected under the fluorescence microscope (Nikon, Japan) and measured by counting cell numbers in at least 3 random fields.

### Sphere formation assay

PTC and ATC cells were resuspended in serum-free RPMI 1640 medium supplemented with B27 (Life Technologies Corporation, USA, 2389253), EGF (SinoBiological, China, 10505-HNAE-100, 20 ng/mL) and bFGF (SinoBiological, China, 10014-HNAE-10, 20 ng/mL). The cells were then seeded in low-adhesion plates (Corning, China, 36421016) and cultured as hanging drops. After 7 days, spheres were collected and their size was measured.

### Flow cytometry analysis

After processed for indicated time, tumor cells were digested into single cell suspensions and then stained with APC anti-human CD133 antibody (Biolegend, USA, 397905) for 30 min at room temperature in dark. The stained cells were washed twice with PBS and resuspended at a volume of 100 µL. Samples were analyzed in the BD-FACSCalibur and Cytoflex LX system. FlowJo^TM^ software (version 10.6.2) was used for data analysis.

### Animal models

BALB/c nude mice (4-6 weeks) were purchased from GemPharmatech Co., Ltd. (Nanjing, China) and randomly assigned to the four groups. 1×10^7^ CAL62 cells were injected subcutaneously into each group of mice. Tumor volume (mm³) was recorded every 4 days and calculated as (length × width²)/2. After 4 weeks, the tumor tissues of sacrificed mice were collected for subsequent analysis. The animal experiments performed in this study were approved by the Ethics Committee of Nanjing Medical University (IACUC-2206017, IACUC-2509040). All procedures were carried out in compliance with the Guide for the Care and Use of Laboratory Animals.

For immunohistochemistry (IHC) staining, collected tumor tissues were first fixed in 10% neutral buffered formalin, then embedded in paraffin and sectioned. IHC staining was performed to detect the expression of the differentiation marker SLC5A5 and the stemness marker CD133. The protein expression levels of these markers were semi-quantitatively evaluated using the IHC score.

### mRNA high-throughput sequencing

Total RNA from stable* IGF2BP2* overexpression TPC1 cells and knockdown CAL62 cells and respective control cells was isolated by TRIzol reagent (Takara, Japan). Quantified by NanoDrop 2000 (Thermo Fisher, USA), 3 μg of RNAs were selected and performed to construct the library with the RNA Sample Pre Kit. Raw data were aligned to hg38 using STAR v2.7.10a. The library construction and next generation sequencing (NGS) were conducted by Sangon Biotech (Shanghai, China).

### RNA immunoprecipitation (RIP) and RIP sequencing

The RIP assay was conducted using the Magna RIP Kit (Millipore, USA) according to the manufacturer's instructions. Briefly, cells were lysed with RIP Buffer supplemented with RNase and protease inhibitors and immunoprecipitated with 5 μg of anti-IGF2BP2 antibody (Proteintech, China, 11601-1-AP) or rabbit IgG (Beyotime, China, A7016) on a 4 °C rocker overnight. Antibody-antigen complexes were then conjugated to Protein A/G Magnetic Beads (Thermo Fisher, USA, 10006D) at 37 °C for 2 h. Following ten washes with RIP buffer, the immunoprecipitated RNA-protein complexes were eluted and isolated with TRIzol reagent (Takara, Japan). Equal mRNA input (500 ng) was used for reverse transcription, qPCR Cq difference (ΔCq = 3.1) reflects template abundance difference only. To visualize PCR products, 10 µL (TPC1) or 5 µL (CAL62) of reaction was loaded on 1.5 % agarose. Bar graphs show within-line IP/IgG ratios. For RIP-sequencing, the co-precipitated mRNA fragment was concentrated for RNA-seq library construction and conducted on the Illumina Hiseq 4000 platform by Personalbio (Shanghai, China).

### CUT&Tag

The CUT&Tag assay was conducted using the NovoNGS® CUT&Tag 4.0 High-Sensitivity Kit for Illumina (Novoprotein, China, N259-YH01) according to the manufacturer's protocols. A total of 1×10^5^ cells were prepared for each sample. Cells were fixed with 1% formaldehyde, permeabilized with 0.2% Digitonin, and incubated with ConA magnetic beads (Novoprotein) for chromatin coupling. Anti-STAT1 primary antibodies (Cell Signaling Technology, USA,14994T) were diluted in antibody buffer (1% BSA, protease inhibitors) and incubated overnight at 4 °C, followed by species-matched secondary antibodies (ChiTag Goat anit-Rabbit IgG antibody, N269) conjugated to Protein A/G (1:500, Thermo Fisher, USA). The ChiTag® pAG-Transposome complex (Novoprotein) was introduced for targeted DNA fragmentation and Illumina adapter ligation in Tagmentation Buffer (0.7 M MgCl₂, 37 °C, 1 h). Reactions were terminated with Stop Buffer, treated with Proteinase K, and purified using AMPure XP beads. Libraries were amplified via PCR (KAPA HiFi HotStart ReadyMix, 12-18 cycles), size-selected with NovoNGS DNA Clean Beads, and quantified using Qubit 4.0 (Thermo Fisher, USA) and an Agilent 2100 Bioanalyzer. DNA integrity was verified by 1.5% agarose gel electrophoresis. Final libraries were sequenced on an Illumina NovaSeq 6000 platform (150-bp paired-end reads) by Personalbio (Shanghai, China).

### Methylated RNA immunoprecipitation (MeRIP) followed by qPCR

Total RNA was isolated using Trizol reagent (TaKaRa, Japan) and fragmented into about 100 nt length verified by 1.5% agarose gel electrophoresis. Then the RNA fragments were immunoprecipitated with m6A antibody (Anti-N6-methyladenosine, Abcam, UK, ab208577) or isotype-matched murine IgG control (Beyotime, China, A7028) in IP buffer (50 mM Tris-HCl pH 7.4, 150 mM NaCl, 1% NP-40) at 4 °C for 2 h. Antibody-RNA complexes were captured by Protein A/G Magnetic Beads (Thermo Fisher, USA, 10006D) through 1 h incubation at 25 °C, followed by five washes with high-salt buffer (50 mM Tris-HCl pH 7.4, 1 M NaCl, 1% NP-40). Immunoprecipitated RNA was eluted using Proteinase K digestion (Thermo Fisher, China, 25530049) and purified via phenol-chloroform extraction. Enrichment of m6A-modified transcripts was quantified by RT-qPCR with methylation-specific primers. The sequence was deposited in Table S2.

### mRNA stability assay

*IGF2BP2* overexpression (TPC1) and knockdown (CAL62) cells were seeded into six-well plates overnight and then incubated with 5 μg/mL actinomycin D (ActD) for 0, 2, 4, 6, 8, and 10 h. Then the relative mRNA levels of* STAT1* were determined by qRT-PCR.

### Luciferase assay

To determine whether IGF2BP2 binds specific m6A sites within the STAT1 transcript, a dual-luciferase reporter assay was performed. Three RNA fragments (STAT1-A, STAT1-B, and STAT1-C), each containing a high-confidence m6A peak predicted by SRAMP (www.cuilab.cn), were synthesized and cloned downstream of the luciferase stop codon in the pGL3-Basic vector by Corues Biotchnology (Nanjing, China). Mutant constructs (STAT1-A-mut and STAT1-B-mut) with A-to-C substitutions within the RRACH motif were generated to disrupt m6A modification. For transfection, CAL62 and TPC1 cells were seeded in six-well plates at 5×10⁵ cells/well and grown to 50% confluence before transfection with the respective reporter plasmids.

To assess STAT1 transcriptional activity, a panel of dual-luciferase reporter plasmids (pGL3-basic, TSHR-luc, SLC26A4-luc, SLC5A5-luc, TPO-luc, PAX8-luc, FOXE1-luc, and NKX2.1-luc) were constructed by Tsingke Biotechnology (Beijing, China). Wild-type TPC1 and CAL62 cells were co-transfected with these reporter plasmids along with a STAT1 expression vector and a Renilla luciferase control. After 48 hours, whole-cell lysates were collected.

Luciferase activity was measured using the Dual-Glo Luciferase Assay System (Promega) 48 hours post-transfection. Firefly luciferase signals were normalized to Renilla activity and expressed relative to negative controls, following the protocol of the Dual Luciferase Reporter Gene Assay Kit (Beyotime, RG027). Primer sequences used for vector construction are provided in Table S3.

### Co-Immunoprecipitation (COIP) assay

COIP was carried out following the manufacturer's protocol (MedChemExpress, USA) with minor modifications. Briefly, human thyroid cancer cells (TPC1 and CAL62) were rinsed twice with ice-cold PBS and lysed in 1 mL of Cell lysis buffer for Western and IP (Beyotime, China, P0013) supplemented with 1% PMSF, 1% phosphatase inhibitor, and 0.1% protease inhibitor. After pre-washing, Protein A/G magnetic beads (Thermo Fisher, USA, 10006D) were used for both preclearance with rabbit IgG (Beyotime, China, A7016) and cell lysate, and for antibody coupling with the specified antibodies, both incubations were performed overnight at 4 °C with gentle mixing. On the following day, the precleared lysate was incubated with the antibody-conjugated beads for 4 h at 4 °C under constant rotation. The immunoprecipitates were collected and washed three times with IP lysis buffer. Proteins were eluted and subjected to Western blot analysis.

### Statistical analysis

Statistical analyses were conducted with GraphPad Prism 10.0, with biological replicates (n = 3) represented as mean ± SD. Pearson's correlation coefficient was calculated to assess linear associations between IGF2BP2 and thyroid differentiation gene expression levels. Intergroup differences were analyzed using unpaired Student's t-test (parametric data) or Mann-Whitney U test (nonparametric data). For multi-group comparisons, one-way ANOVA with Tukey's post hoc test was applied. *P* < 0.05 was considered statistically significant.

## Results

### IGF2BP2 expression was upregulated in THCA, especially in ATC, indicating a poor prognosis

**Figure 1:**
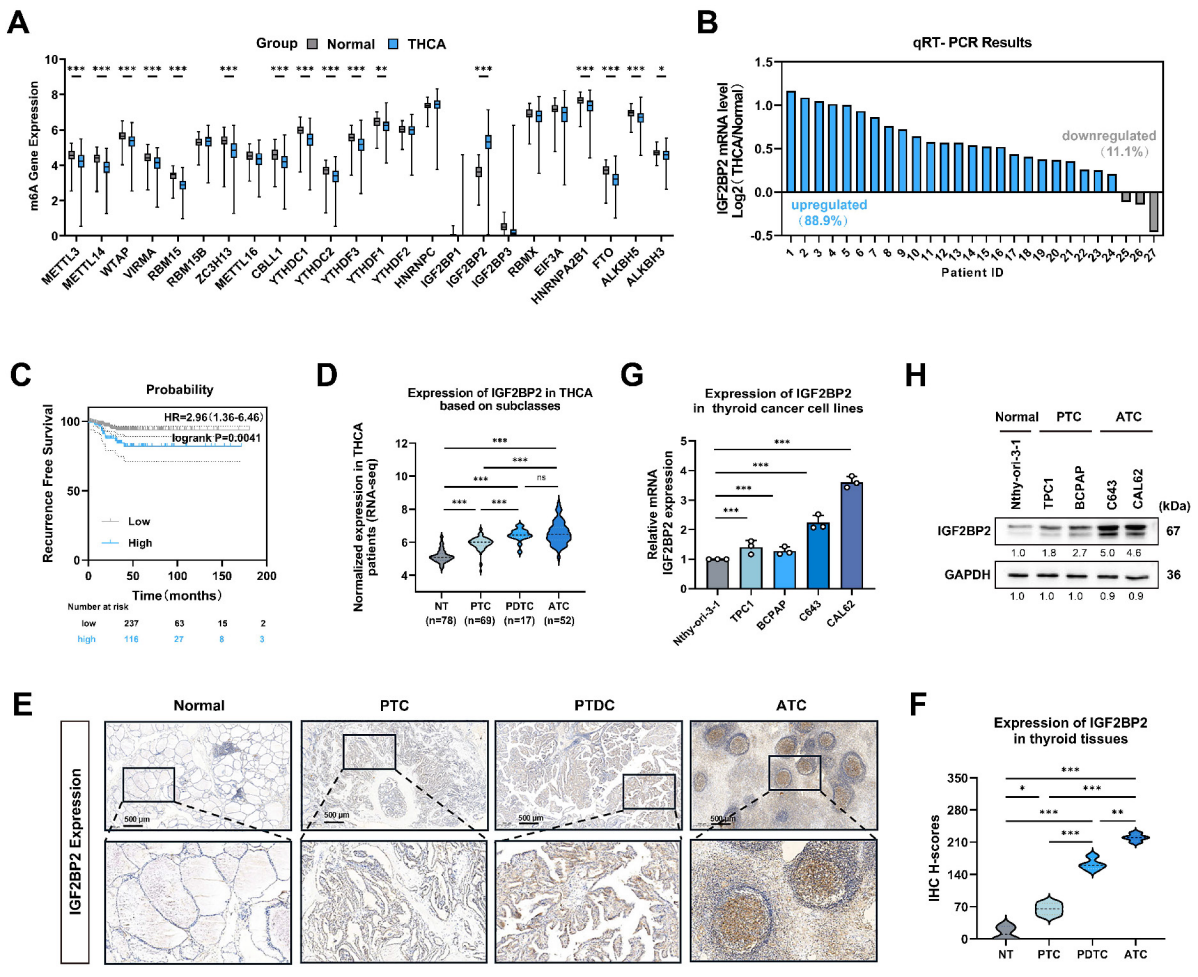
**IGF2BP2 was aberrantly expressed in THCA, especially in ATC, and related to poor prognosis.** (A) Relative expression of 'm6A regulators' in thyroid carcinoma and normal thyroid tissue in the TCGA-THCA databases. (B) Quantitative real-time PCR was used to measure the mRNA level of *IGF2BP2* in normal and thyroid cancer tissues. (C) Kaplan-Meier survival analysis performed using the KM Plotter online tool showed that upregulation of IGF2BP2 was significantly associated with shorter RFS in THCA patients. (D) The expression of IGF2BP2 in human normal thyroid cells, PTC cells, PDTC cells, and ATC cells was mined from the GEO datasets (GSE29265, GSE65144, GSE76039, and GSE33630). (E) IGF2BP2 expression in normal thyroid tissues, PTC tissues, PDTC and ATC tissues was observed by immunohistochemistry staining. Scale bars, 500 μm. (F) IHC H-scores for IGF2BP2 expression are quantified and presented as the mean ± SD in the adjacent violin graphs. Scale bar, 500 μm (applicable to all images). (G-H) The expression levels of IGF2BP2 were examined by qRT-PCR (G), and western blot (H) in human normal thyroid cells, PTC cells, and ATC cells. Intergroup differences were analyzed using unpaired Student's t-test (parametric data) or Mann-Whitney U test (nonparametric data). For multi-group comparisons, one-way ANOVA with Tukey's post hoc test was applied. All data: mean ± SEM; * *P* < 0.05, ** *P* < 0.01, *** *P* < 0.001).

To investigate the role of 'm6A regulators' in thyroid cancer progression, we analyzed bulk RNA-seq data from 512 thyroid carcinoma (THCA) and 59 normal thyroid tissue samples sourced from TCGA datasets. Among the 'm6A regulators' examined, *IGF2BP2* mRNA exhibited the most significant dysregulation compared to normal tissues (Fig. 1A). Consistent with these findings, qRT-PCR analysis of THCA patient samples confirmed significantly elevated IGF2BP2 expression in tumor tissues compared to matched adjacent normal tissues (Fig. 1B).

Using the KM plotter online tool for survival analysis, we found that high IGF2BP2 expression was significantly associated with shorter recurrence-free survival (RFS) (*P* = 0.0041) (Fig. 1C). Similarly, IGF2BP2 expression was significantly upregulated in undifferentiated and poorly differentiated thyroid carcinomas within the GEO database (Fig. 1D), histological subtypes known for poor survival and treatment resistance. This progressive elevation of IGF2BP2 expression was further validated by immunohistochemistry (IHC) staining, which revealed significantly increased IGF2BP2 levels in PTC, PDTC and ATC compared to normal thyroid tissues (Fig. 1E-F). We next assessed IGF2BP2 relative expression by qRT-PCR in a panel of cell lines: the normal thyroid follicular epithelial cell line (Nthy-ori 3-1), PTC cell lines (TPC1, and BCPAP), and ATC cell lines (C643, and CAL62) (Fig. 1G). Concordant with mRNA levels, Western blotting confirmed correspondingly elevated IGF2BP2 protein levels across these distinct cell lines (Fig. 1H). Thus, these findings establish IGF2BP2 as markedly upregulated in thyroid carcinoma, with highest expression in aggressive subtypes, and significantly associated with poor clinical outcomes.

### IGF2BP2 promoted the proliferation, and differentiation courses of THCA

**Figure 2:**
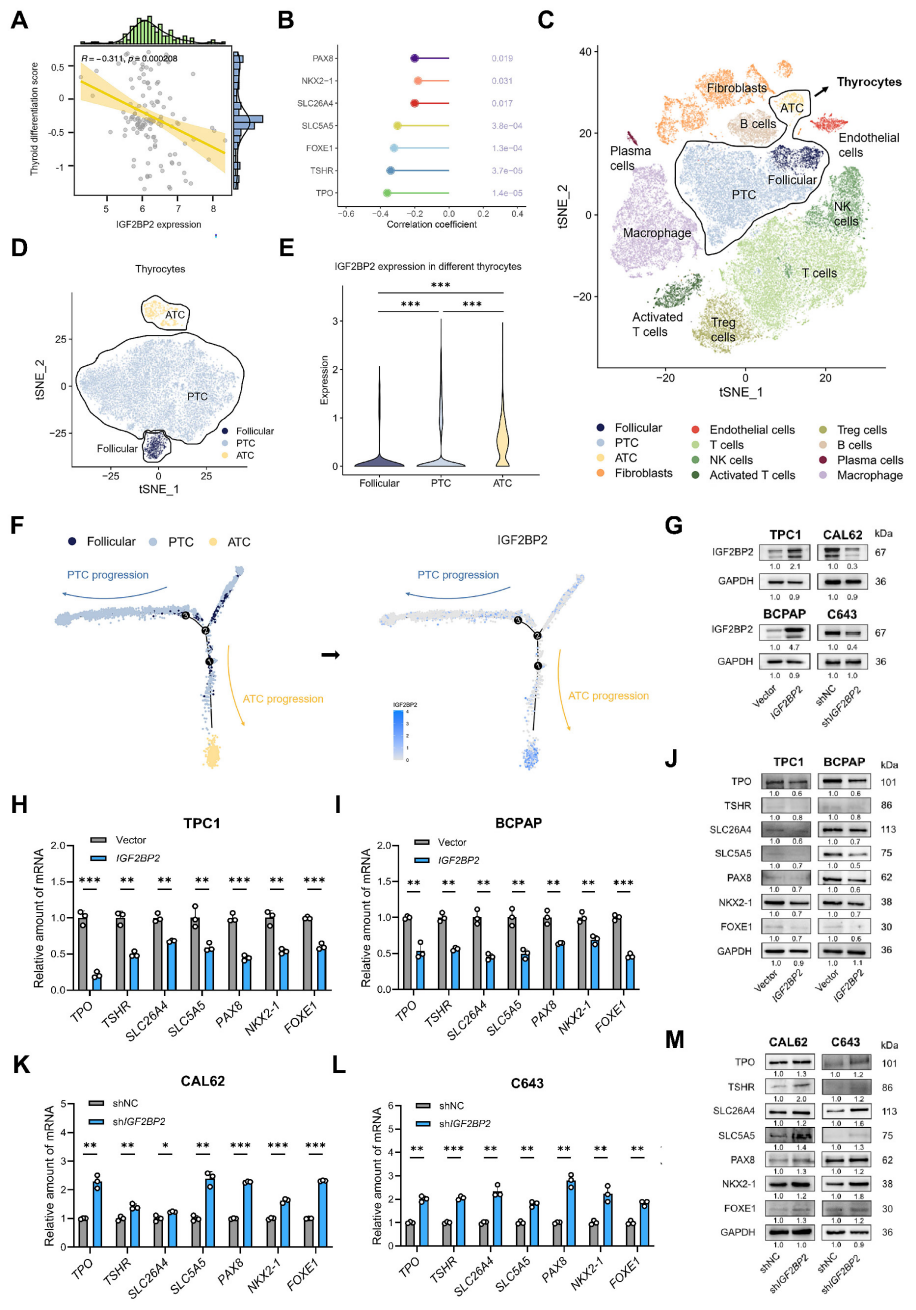
**IGF2BP2 was closely correlated with dedifferentiation of THCA.** (A) The correlation between IGF2BP2 expression and TDS. (B) The lollipop chart summarizes the correlation between IGF2BP2 and thyroid differentiation genes in GEO datasets. (C) tSNE demonstrates the 12 cell clusters using scRNA-seq data. (D) Identification of thyroid cells using scRNA-seq data. (E) The expression pattern of *IGF2BP2* in identified normal follicular epithelium, PTC and ATC cells. (F) Single-cell trajectory analysis depicting the expression of IGF2BP2 during thyroid cancer progression. Color scale represents expression level. (G) The efficiency of stable *IGF2BP2* overexpression and knockdown cell lines verified by Western blot. (H-I) The mRNA levels of differentiation markers were evaluated by RT-qPCR in *IGF2BP2*-overexpressed PTC cell lines. (J) The changes of differentiation markers in PTC cell lines determined by Western blot. (K-L) The mRNA levels of differentiation markers were evaluated by RT-qPCR in *IGF2BP2* knockdown ATC cell lines. (M) The changes of differentiation markers in ATC cell lines were determined by Western blot. *P* values were determined using a two-tailed unpaired Student's test (* *P* < 0.05, ** *P* < 0.01, *** P < 0.001).

Given the progressively increasing expression levels of IGF2BP2 across normal thyroid, PTC, PDTC, and ATC cell lines and tissues (Fig. 1D-G), we hypothesized that IGF2BP2 may contribute to the dedifferentiation phenotype in thyroid carcinoma. To test this hypothesis, we employed the thyroid differentiation score (TDS)^8^, a metric integrating the expression of 16 thyroid metabolism and function-associated genes, to assess the differentiation status of tumors from 138 THCA patients within the GEO database. Consistent with our hypothesis, analysis revealed a significant negative correlation between IGF2BP2 expression and TDS (Fig. 2A), indicating that higher IGF2BP2 levels are associated with poorer differentiation. Further interrogation of the GEO database demonstrated significant negative correlations between IGF2BP2 expression and key thyroid differentiation markers, including TPO, TSHR, SLC26A4, SLC5A5, PAX8, FOXE1, and NKX2.1 (Fig. 2B). To delineate the dynamic association of IGF2BP2 with dedifferentiation trajectories, we leveraged single-cell RNA sequencing (scRNA-seq) data (Fig. 2C-D, Fig. S1A). Based on established models of follicular epithelial cell differentiation which bifurcate into PTC-derived PTC cells (pPTC) and ATC-derived PTC cells (aPTC)^4^, pseudotime trajectory analysis revealed significant enrichment of IGF2BP2 expression specifically along the aPTC differentiation path (Fig. 2E-F).

To directly investigate the functional role of* IGF2BP2* in THCA dedifferentiation, we overexpressed *IGF2BP2* in two PTC cell lines (TPC1, and BCPAP) exhibiting relatively low endogenous* IGF2BP2* levels. Conversely, we knocked down IGF2BP2 expression in ATC cell lines (CAL62, and C643) characterized by high IGF2BP2 expression (Fig. 2G, Fig. S1B-C). We first assessed cellular proliferation. CCK-8 assays and EdU staining collectively demonstrated that* IGF2BP2* overexpression promoted PTC cell proliferation Fig. S1D-G), while *IGF2BP2* knockdown significantly impaired ATC cell growth (Fig. S1H-K).

Given that cancer progression involves gradual dedifferentiation and acquisition of stem cell-like properties(32, we next evaluated the impact of IGF2BP2 manipulation on differentiation marker expression. qRT-PCR analysis revealed that* IGF2BP2* overexpression in TPC1 and BCPAP cells significantly downregulated the transcriptional levels of *TSHR*, *SLC26A4*, *SLC5A5*, *TPO*, *PAX8*, *FOXE1*, and* NKX2.1* (Fig. 2H-I). Concordantly, Western blotting demonstrated reduced protein levels of these seven differentiation markers (Fig. 2J). Conversely, *IGF2BP2* knockdown in CAL62 and C643 cells significantly upregulated the mRNA levels of these differentiation-related molecules (Fig. 2K-L) and increased their protein levels (Fig. 2M). Collectively, these findings uncover that IGF2BP2 expression inversely correlates with thyroid differentiation status in clinical cohorts and directly drives dedifferentiation *in vitro*, repressing key thyroid-specific markers at both mRNA and protein levels while promoting cellular proliferation.

### IGF2BP2 sustained the stemness in thyroid carcinoma

**Figure 3:**
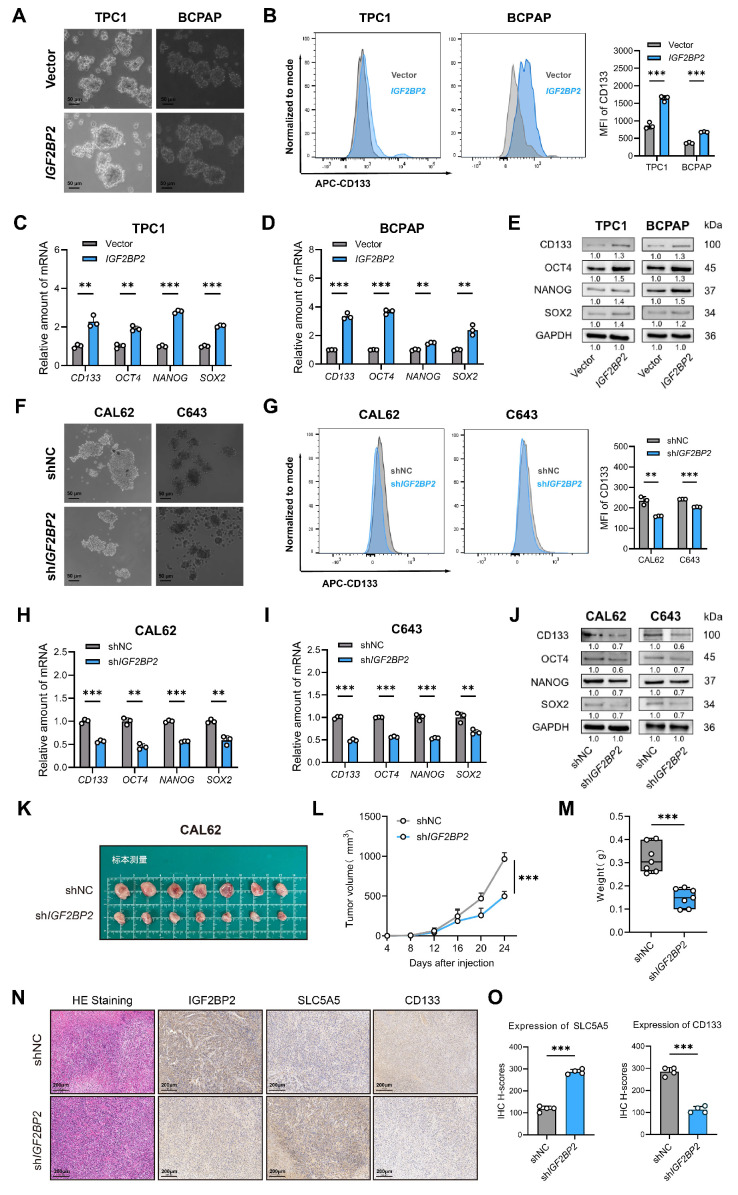
** IGF2BP2 maintained stemness of THCA.** (A) *IGF2BP2* overexpression promoted sphere formation ability of TPC1 and BCPAP cells. (Scale bar, 50 μm). (B) Flow cytometry analysis and mean fluorescence intensity of CD133 expression following *IGF2BP2* overexpression in TPC1 and BCPAP. The mRNA (C-D) and protein (E) levels of stemness markers were assessed by RT-qPCR and western blot following *IGF2BP2* overexpression in TPC1 and BCPAP, respectively. (F)* IGF2BP2* knockdown inhibited sphere formation ability of CAL62 and C643 cells. (Scale bar, 50 μm). (G) Flow cytometry analysis and mean fluorescence intensity of CD133 expression upon *IGF2BP2* knockdown in CAL62 and C643. The mRNA (H-I) and protein (J) levels of stemness markers were assessed by RT-qPCR and Western blot following *IGF2BP2* knockdown in CAL62 and C643, respectively. (K-M) The tumor growth curve, and tumor weight of CAL62 xenograft in nude mice. (N) Representative immunohistochemical (IHC) staining of IGF2BP2, SLC5A5, and CD133 in xenograft tumors from each experimental group. Scale bar, 200 μm (applicable to all images). (O) IHC H-scores for SLC5A5 and CD133 expression are quantified and presented as the mean ± SD in the adjacent bar graphs. *P* values were determined using a two-tailed unpaired Student's test (* *P* < 0.05, ** *P* < 0.01, *** P < 0.001).

During oncogenesis, differentiated cells undergo phenotypic regression, acquiring primitive progenitor and stem-like features that drive tumor aggressiveness^33^. Given the observed association between dedifferentiation and enhanced stemness, we hypothesized that IGF2BP2 concurrently promotes cellular dedifferentiation and augments tumor stemness in thyroid carcinoma. Subsequent experimental validation confirmed this association. Given that CD133 is a recognized marker of thyroid cancer stem cells (CSCs)^16^, we evaluated stemness properties in engineered cell models. Overexpression of* IGF2BP2* notably enhanced the sphere-forming capacity of PTC cell lines, accompanied by a significant increase in both the CD133⁺ cell population and the mean fluorescence intensity (MFI) of CD133 (Fig. 3A-B). In contrast, knockdown of *IGF2BP2* markedly impaired spheroid formation and reduced CD133 expression, as evidenced by decreased CD133 MFI in ATC-derived subclones (Fig. 3F-G). Consistent with these findings, qRT-PCR analysis demonstrated that *IGF2BP2* overexpression significantly upregulated the mRNA levels of key stemness markers, including *CD133, NANOG, OCT4, and SOX2* (Fig. 3C-D). Immunoblot analysis further confirmed the increase in their protein levels (Fig. 3E). Reciprocally, silencing* IGF2BP2* expression led to a significant downregulation of the mRNA levels of these markers (Fig. 3H-I) and reduced their protein levels (Fig. 3J), indicating diminished stemness. To evaluate the functional role of IGF2BP2 in thyroid cancer progression *in vivo*, we established xenograft models using* IGF2BP2*-knockdown CAL62 cells and control cells. *IGF2BP2* inhibition significantly impaired the growth of transplanted tumors in immunodeficient mice (Fig. 3K-M). Immunohistochemical analysis of xenograft tumors revealed that *IGF2BP2* knockdown promoted redifferentiation, as evidenced by a concomitant significant increase in the differentiation marker SLC5A5 (NIS) with a marked reduction in the cancer stem cell marker CD133, compared to shNC controls (Fig. 3N-O). Generally, our data highlighted a crucial role for IGF2BP2 in driving both the dedifferentiation program and the maintenance of stemness properties in thyroid carcinoma at both transcriptional and translational levels.

### Identification of IGF2BP2 targets mediating thyroid cancer dedifferentiation

**Figure 4:**
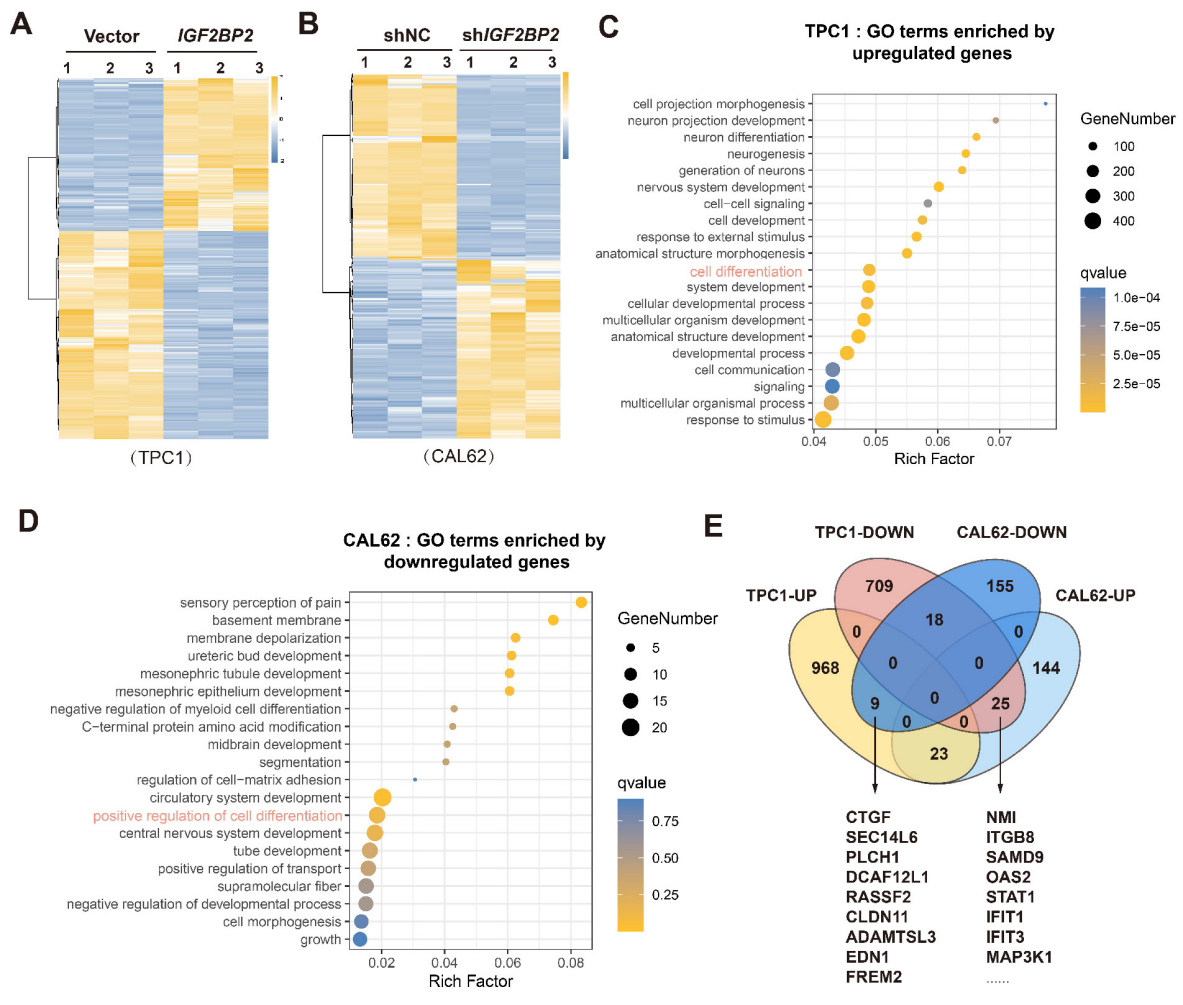
** Identification of the IGF2BP2 targets in thyroid cancer in various differentiation states.** (A) Heatmap showing differentially expressed genes (DEGs) between Vector group and *IGF2BP2* group in TPC1 cells as determined by RNA-sequencing. (B) Heatmap depicting DEGs between shNC group and sh*IGF2BP2* group in CAL62 cells identified by RNA-sequencing. (C-D) GO enrichment analysis of DEGs. (E) Transcriptomic intersection analysis reveals the potential targets of IGF2BP2 in dedifferentiation.

To elucidate the mechanism by which IGF2BP2 promotes thyroid cancer dedifferentiation, we performed RNA sequencing (RNA-seq) on TPC1 cells overexpressing *IGF2BP2* (TPC1-OE) and CAL62 cells with *IGF2BP2* knockdown (CAL62-KD). *IGF2BP2* overexpression in TPC1 cells significantly altered the transcriptome, with 1000 genes upregulated and 752 genes downregulated (Fig. 4A). Conversely, *IGF2BP2* knockdown in CAL62 cells resulted in significant upregulation of 192 genes and downregulation of 182 genes (Fig. 4B). Gene ontology (GO) analysis of these differentially expressed genes (DEGs) revealed significant enrichment in pathways related to cell differentiation (Fig. 4C-D).

To identify potential downstream effectors of IGF2BP2-driven dedifferentiation, we performed an intersection analysis of the DEGs across these two contrasting thyroid cancer models, TPC1-OE and CAL62-KD. This analysis identified 9 consistently upregulated targets (elevated in TPC1-OE and reduced in CAL62-KD) and 23 consistently downregulated targets (reduced in TPC1-OE and elevated in CAL62-KD) (Fig. 4E). These high-confidence IGF2BP2-regulated genes represented prime candidates mechanistically linking IGF2BP2 to the promotion of dedifferentiation and stemness maintenance in thyroid cancer.

### IGF2BP2 suppressed STAT1 expression via m6A modification to promote dedifferentiation

**Figure 5:**
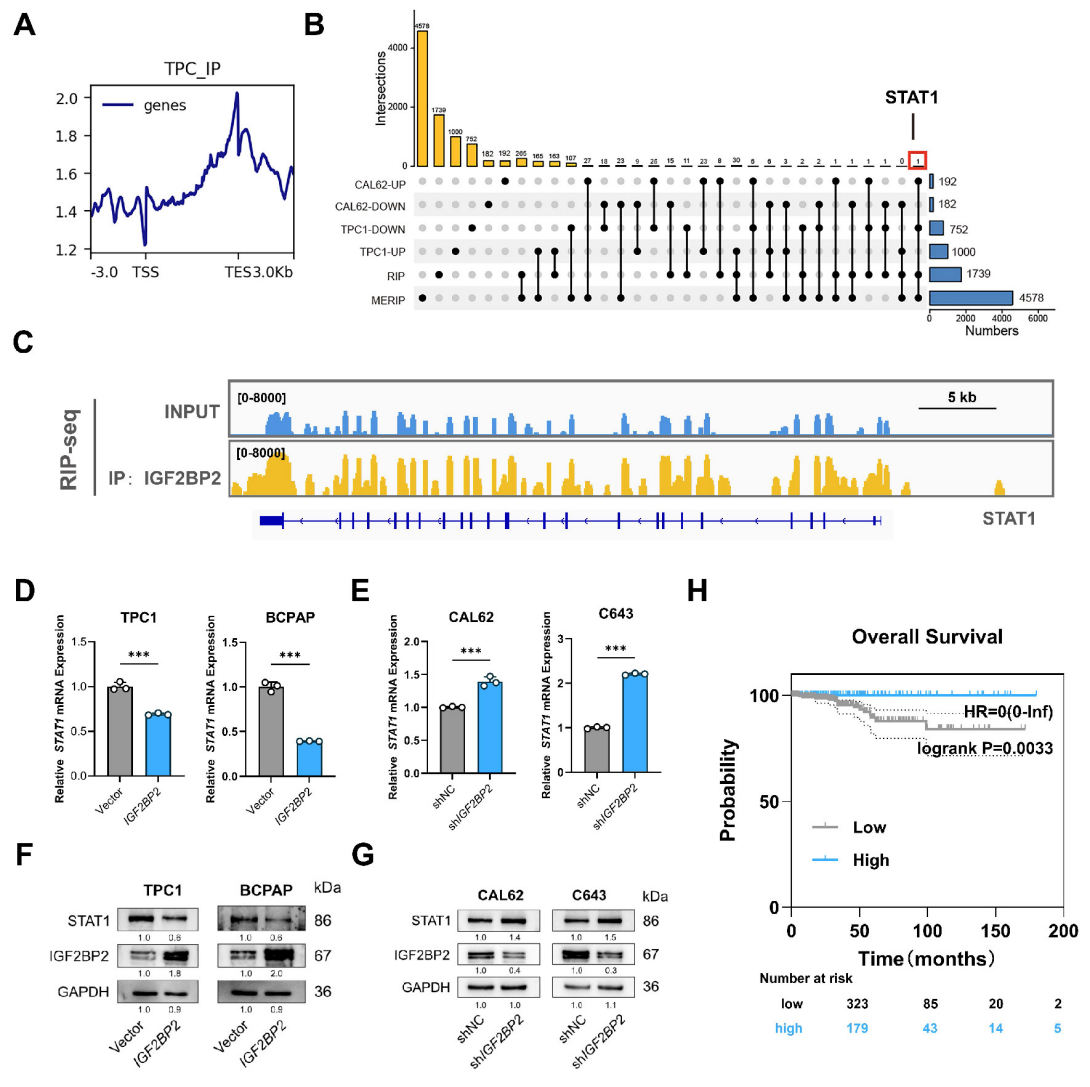
** STAT1 was a m6A target of IGF2BP2 in thyroid cancer associated with the dedifferentiation.** (A) Distribution of IGF2BP2-binding peak of RIP-seq. (B) The upset plot shows the intersection of RNA-seq, RIP-seq, and MERIP seq (GSE199205). (C) IGF2BP2 binding peaks in *STAT1* transcripts visualized by IGV. Relative RNA level of *STAT1* in PTC following* IGF2BP2* overexpression (D) and ATC upon *IGF2BP2* knockdown (E). (F) Western blot detected the protein level of STAT1 in PTC cells transfected with overexpressing lentiviruses carrying IGF2BP2. (G) The protein levels of STAT1 were measured by western blot analysis in ATC cells transfected with lentiviruses carrying sh-*IGF2BP2*. (H) Kaplan-Meier analysis of overall survival of THCA patients using the KM Plotter online tool.* P* values were determined using a two-tailed unpaired Student's test (* *P* < 0.05, ** *P* < 0.01, *** P < 0.001).

IGF2BP2, an m6A reader known to stabilize mRNAs and enhance translation via its K Homology (KH) domains^26^. To identify direct IGF2BP2 targets in thyroid cancer, we performed RIP-seq in TPC1 cells, revealing 3377 binding peaks across 1739 genes, predominantly localized near transcription termination sites (TES). Intersection of RIP-seq targets with m6A-modified transcripts (from MERIP-seq data: GSE199205) identified 265 m6A-modified IGF2BP2-bound transcripts. Among these, STAT1 emerged as the sole IGF2BP2 m6A target linked to dedifferentiation (Fig. 5B), with IGF2BP2 binding enrichment visualized in its coding sequence (CDS) region (Fig. 5C). Functional assessment demonstrated that *IGF2BP2* overexpression in PTC cells suppressed both *STAT1* mRNA and protein levels, while *IGF2BP2* knockdown in ATC cells significantly upregulated both *STAT1* mRNA level and STAT1 protein level (Fig. 5D-G). Clinically, high *STAT1* mRNA expression correlated with significantly longer overall survival in thyroid cancer patients (*P*=0.0033, Fig. 5H). These data establish STAT1 as a direct m6A-modified target of IGF2BP2 in thyroid cancer cells.

### STAT1 transcriptionally activated thyroid differentiation genes

STAT1, recognized as a tumor suppressor and regulator of metabolic processes^34^, functions as a canonical transcription factor. We hypothesized that STAT1 directly governs differentiation-associated genetic programs, particularly those controlling thyroid-specific metabolism and function. Initial* STAT1* knockdown in TPC1 cells (reducing STAT1 mRNA and protein levels) and overexpression in CAL62 cells (increasing STAT1 mRNA and protein levels) confirmed that STAT1 levels did not affect IGF2BP2 mRNA or protein expression Fig. S2A-C). Subsequent functional assays demonstrated STAT1's tumor-suppressive role: *STAT1* knockdown promoted, while overexpression inhibited thyroid cancer cell proliferation (Fig. S2D-I).

**Figure 6:**
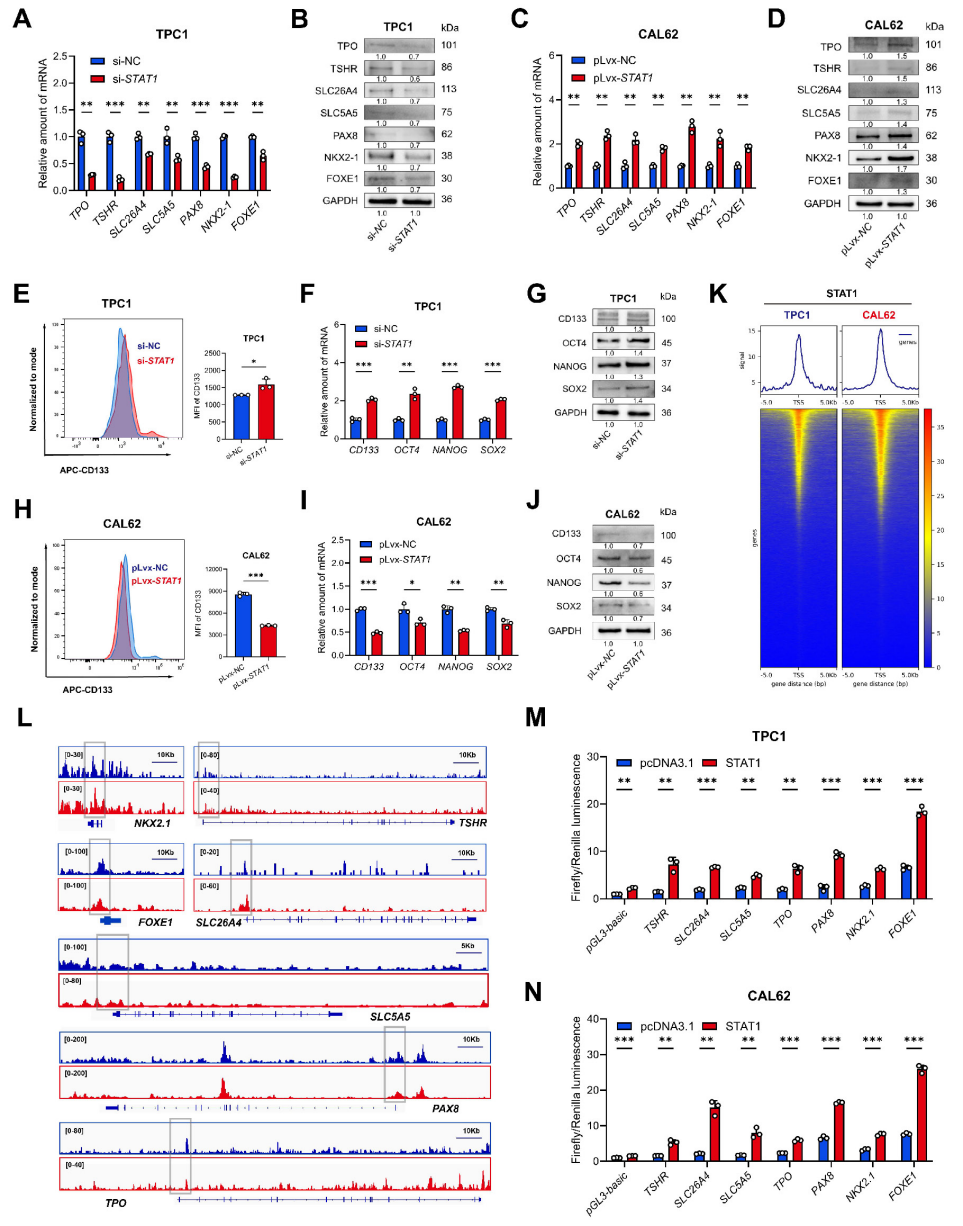
** STAT1 activated the transcription of thyroid differentiation genes in thyroid cancer and mediated the differentiation and stemness.** (A-B) Relative mRNA (A) and protein (B) level of thyroid differentiation-related genes measured by qRT-PCR in *STAT1*-knockdown TPC1 cells. (C-D) The mRNA (C) and protein (D) variation of dedifferentiation molecules was measured by western blot in CAL62 cells. (E) Layout and MFI of CD133 expression in TPC1 cells determined by flow cytometry. (F-G) The transcript (F) and protein (G) levels of stemness markers in si-*STAT1* TPC1 cells. (H) Layout and MFI of CD133 expression in CAL62 cells determined by flow cytometry. (I-J) The transcript (I) and protein (J) levels of stemness markers in *STAT1*-OE CAL62 cells. (K) CUT&Tag was performed with STAT1 antibody in TPC1 and CAL62 cells. Heatmap showing the genomic distribution of STAT1 flanking TSSs in TPC1 and CAL62 cells. (L) IGV tracks for thyroid differentiation genes from CUT&Tag. (M-N) Dual-luciferase reporter assay of p*GL3-basic, TSHR, SLC26A4, SLC5A5, TPO, PAX8, FOXE1,* and *NKX2.1* promoter activity driven by STAT1 or pcDNA3.1 transfection in wild-type TPC1 and CAL62 tumor cells. Data are relative to Renilla luciferase activity. n = 3. *P* values were determined using a two-tailed unpaired Student's test (* *P* < 0.05, ** *P* < 0.01, *** P < 0.001).

Crucially, *STAT1* silencing in TPC1 cells significantly downregulated both mRNA and protein levels of key thyroid differentiation genes involved in metabolism and function (Fig. 6A-B). Conversely, *STAT1* overexpression in CAL62 cells robustly upregulated both mRNA and protein levels of these genes (Fig. 6C-D). To evaluate the role of STAT1 in stemness regulation, functional assays revealed that *STAT1* knockdown increased CD133 membrane expression. Conversely, *STAT1* overexpression sharply reduced CD133 levels, as confirmed by flow cytometry (Fig. 6E, H). Concordantly, mRNA levels of CSC markers were inversely correlated with *STAT1* mRNA expression, while protein levels of CSC markers were inversely correlated with STAT1 protein expression (Fig. 6F-G, I-J). Collectively, these results established STAT1 as a potent negative regulator of thyroid cancer dedifferentiation and stemness.

To investigate the underlying transcriptional mechanism, we performed CUT&Tag assays (a genome-wide immunotethering technique) using a ChIP-grade STAT1 antibody in TPC1 and CAL62 cells. Genome-wide distribution analysis using deepTools revealed that 31.75% (TPC1) and 33.57% (CAL62) of STAT1 binding peaks were localized within promoter regions (±3 kb from TSS) (Fig. 6K, Fig. S2J-K). Visualization of STAT1 binding signals using the Integrative Genomics Viewer (IGV) detected significant STAT1 enrichment around transcription start sites (TSS) of thyroid differentiation genes in both models (Fig. 6L). Notably, the substantial overlap of STAT1 binding peaks between PTC and ATC cells (Fig. S2L) further supported functional conservation. To determine whether STAT1 directly transactivates the promoters of thyroid differentiation related genes, dual-luciferase reporter assays were carried out using promoter sequence of *pGL3-basic, TSHR, SLC26A4, SLC5A5, TPO, PAX8, FOXE1, and NKX2.1*. Transient overexpression of STAT1 in TPC1 and CAL62 cells resulted in significant upregulation of luciferase activity, demonstrating the functional enhancement of these promoters by STAT1 (Fig. 6M-N). In summary, these findings demonstrate that STAT1 was directly bound to promoter-proximal regions of differentiation-related genes, positioning it as a master transcriptional regulator of thyroid cell identity.

### IGF2BP2 destabilized *STAT1* mRNA in an m6A-dependent manner

**Figure 7:**
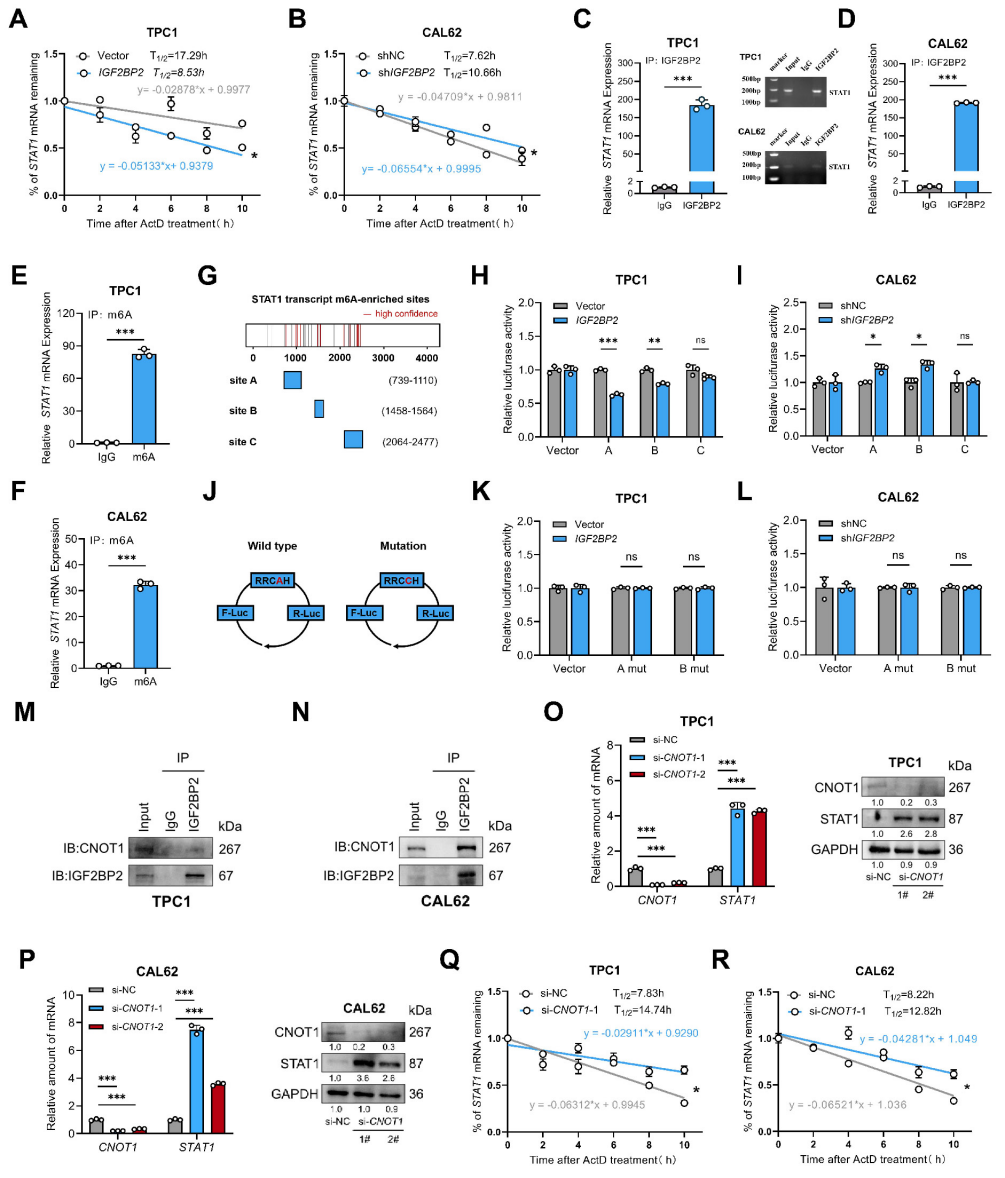
** IGF2BP2 regulated stabilization of STAT1 transcript via an m6A-dependent manner.** (A-B) TPC1-OE and CAL62-KD cells were treated with 5 μg/mL actinomycin D (ActD) for 0, 2, 4, 6, 8, and 10 h, followed by RT-qPCR. (C-F) TPC1 and CAL62 cell lysates were immunoprecipitated with IGF2BP2 or m6A antibody and control immunoglobulin G (IgG) to detect *STAT1* mRNA expression and validated by agarose electrophoresis. (G) Sketch map shows the m6A -enriched sites of* STAT1* transcript. (H-I) Dual-luciferase assay of STAT1 reporter activity driven by STAT1-A, B, and C transfection in vector and* IGF2BP2* overexpression TPC1 cells as well as* IGF2BP2* knockdown CAL62 cells. Data are relative to Renilla luciferase activity. n = 3. (J) Sketch map shows the dual-luciferase reporter plasmid construction for *STAT1* m6A site validation. (K-L) Dual-luciferase assay of *STAT1* reporter activity driven by STAT1-A mutant, and B mutant transfection in vector and *IGF2BP2* overexpression TPC1 cells as well as* IGF2BP2* knockdown CAL62 cells. Data are relative to Renilla luciferase activity. n = 3. (M-N) Co-IP and Western blot analysis of TPC1 (M) and CAL62 (N) cells demonstrate that IGF2BP2 is physically associated with CNOT1. (O-P) The relative *STAT1* mRNA abundance and protein expression in si-*CNOT1* TPC1 (O) and CAL62 cells (P). (Q-R) TPC1 (Q) and CAL62 (R) si-*CNOT1* cells were treated with 5 μg/mL actinomycin D (ActD) for 0, 2, 4, 6, 8, and 10 h, followed by RT-qPCR. *P* values were determined using a two-tailed unpaired Student's test (* *P* < 0.05, ** *P* < 0.01, *** P < 0.001). * Method note: TPC1 and CAL62 RIPs were performed in separate batches with different starting cell numbers (10 × 10⁶ vs 6 × 10⁶). Equal amounts of eluted mRNA (500 ng) were reverse-transcribed; qPCR revealed 3.1 cycles higher Cp in CAL62, indicating ~8.6-fold lower template abundance. 10 µL PCR product was loaded for TPC1 and 5 µL for CAL62 (same cycle number). Bar graphs display within-cell-line IP/IgG enrichment ratios, absolute abundance cannot be compared across lines.

Having established IGF2BP2 as a negative regulator of STAT1 expression, we next investigated the underlying mechanism. As an m6A reader known to modulate mRNA stability and translation(26, IGF2BP2 may directly interact with *STAT1* mRNA. To test this mechanism, control and *IGF2BP2* overexpression or knockdown thyroid cancer cells were treated with 5 μg/mL RNA synthesis inhibitors actinomycin (ActD). As expected, the mRNA stability assays revealed that *STAT1* mRNA half-life was shortened upon *IGF2BP2* overexpression and prolonged following* IGF2BP2* knockdown in CAL62 cells (Fig. 7A-B). Direct binding of IGF2BP2 to *STAT1* mRNA in both PTC and ATC cell lines was confirmed by RIP assay, demonstrating the consistency of the IGF2BP2-STAT1 axis across thyroid cancer subtypes (Fig. 7C-D). Furthermore, MeRIP assays verified that the binding of IGF2BP2 to *STAT1* mRNA was m6A-dependent (Fig. 7E-F).

To functionally link specific m6A sites to IGF2BP2-mediated regulation, we constructed luciferase reporter plasmids harboring high-confidence m6A motifs (sites A, B, C) within the *STAT1* sequence, predicted by SRAMP (www.cuilab.cn), with pGL3 as a negative control (Fig. 7G). Dual-luciferase reporter assays showed that *IGF2BP2* overexpression significantly decreased the activity of reporters containing sites A and B (Fig. 7H), whereas *IGF2BP2* knockdown in CAL62 cells increased their activity (Fig. 7I). Reporter activity for site C remained unchanged. We further introduced A-to-C mutations within the RRACH motifs of sites A and B to disrupt m6A modification (Fig. 7J). Notably, these mutations abolished the regulatory effects of IGF2BP2. The luciferase activities of the mutant reporters were no longer responsive to *IGF2BP2* overexpression in TPC1 cells (Fig. 7K) or to knockdown in CAL62 cells (Fig. 7L). To assess the structural context of the identified m6A sites, we predicted the secondary structure of both the full-length *STAT1* mRNA and the corresponding isolated fragments. While some local structural variations were noted between the contexts Fig. S3, our functional luciferase assays demonstrate that the primary sequence of sites A and B is sufficient to confer IGF2BP2-dependent regulation in cells.

Overall, these results demonstrate that IGF2BP2 recognizes m6A modifications (specifically at sites A and B) on *STAT1* mRNA, leading to its destabilization and consequently, suppression of STAT1 protein expression.

The CCR4-NOT deadenylase complex, a key mediator of cytoplasmic mRNA decay, promotes RNA degradation by shortening the poly(A) tail to initiate 3′-5′ exonucleolytic decay. Given prior evidence that IGF2BP2 recruits this complex via its scaffold subunit CNOT1 to destabilize target mRNAs like *Fzd8* and *PR* 35,36, we hypothesized that IGF2BP2 employs the same mechanism for *STAT1* mRNA. To test this, we first confirmed the specific interaction between IGF2BP2 and CNOT1 by co-immunoprecipitation in TPC1 and CAL62 cells (Fig. 7M-N). Supporting the functional relevance of this interaction, *CNOT1* knockdown significantly increased both the mRNA and protein abundance of STAT1 (Fig. 7O-P). Furthermore, *CNOT1* knockdown markedly extended the half-life of *STAT1* mRNA (Fig. 7Q-R). Collectively, these results demonstrate that IGF2BP2 recruits the CCR4-NOT complex through CNOT1 to facilitate the deadenylation and decay of *STAT1* transcripts.

### STAT1 replenishment attenuated IGF2BP2-driven loss of differentiation in thyroid cancer models

**Figure 8:**
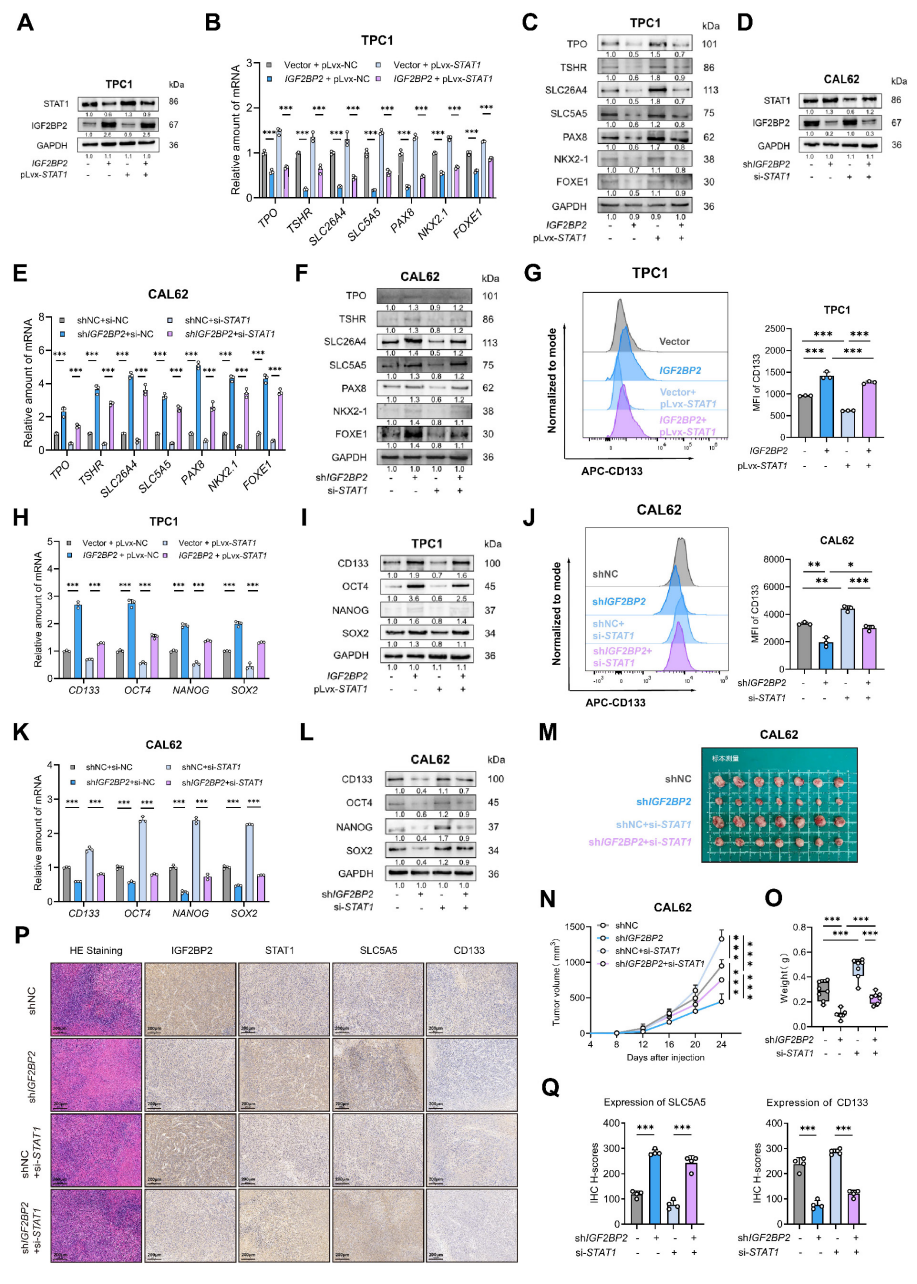
** STAT1 reversed the vicious dedifferentiation, and stemness promoted by IGF2BP2 in thyroid cancer.** (A) The TPC1-OE cells were transfected with pLvx-STAT1 plasmids confirmed by Western blotting. (B-C) Thyroid differentiation factor expressions were measured by qRT-PCR (B) and Western blot (C) in TPC1 cell lines. (D) CAL62-KD cells were interfered with si-*STAT1*, the transfection efficiency was confirmed by Western blotting. (E-F) Thyroid differentiation factor expressions were measured by qRT-PCR (E) and Western blot (F) in TPC1 cell lines. (G) The CSCs CD133 features were measured by were assessed by flow cytometry in TPC1 cells. (H-I) The RNA (H) and protein (I) levels of stemness markers in TPC1 cells. (J) The CSCs CD133 features were measured by were assessed by flow cytometry in CAL62 cells. (K-L) The mRNA (K) and protein (L) levels of CSCs markers were measured by western blot analysis. (M-O) Growth curve and tumor weight of subcutaneous xenografts models with CAL62 cells. (P) Representative immunohistochemical (IHC) staining of IGF2BP2, STAT1, SLC5A5, and CD133 in xenograft tumors from each experimental group. Scale bar, 200 μm (applicable to all images). (Q) IHC H-scores for SLC5A5 and CD133 expression are quantified and presented as the mean ± SD in the adjacent bar graphs. *P* values were determined using a two-tailed unpaired Student's test (* *P* < 0.05, ** *P* < 0.01, *** P < 0.001).

To determine if STAT1 is the key downstream effector mediating IGF2BP2's pro-dedifferentiation effects, we performed rescue experiments. We overexpressed* STAT1* in *IGF2BP2*-overexpressing TPC1 cells and knocked down *STAT1* in *IGF2BP2*-depleted CAL62 cells. Transfection efficiency was confirmed at mRNA and protein levels Fig. S4A-B, Fig. 8A,D). *IGF2BP2* overexpression promoted the proliferation, suppressed thyroid differentiation markers (at mRNA and protein levels), and enhanced stemness properties (CD133⁺ population and CSC marker expression) in TPC1 cells, concomitant *STAT1* overexpression significantly reversed these malignant phenotypes (Fig. 8B-C, G-I, Fig. S4E-G). Likewise, the interfered *STAT1* expression effectively counteracted the impact of *IGF2BP2* inhibition in CAL62 cells, restoring proliferation (Fig. S4H-J) and validating that STAT1 mediates the function of IGF2BP2 in promoting thyroid cancer dedifferentiation (Fig. 8E-F) and CSCs features (Fig. 8J-L). Additionally, further rescue experiments* in vivo* were conducted. In xenograft models, the impaired tumor growth induced by* IGF2BP2* inhibition could be reinstated by si-*STAT1* (Fig. 8M-O). IHC staining of xenograft tumors further verified the rescue effect (Fig. 8P-Q). Taken together, our results showed that STAT1 was a dominant contributor responsible for the pro-dedifferentiation and tumor-promoting effects driven by IGF2BP2 in thyroid cancer.

## Discussion

The progression of advanced thyroid cancer is frequently driven by the acquisition of stem-like properties and dedifferentiation, yet the molecular drivers governing this phenotypic plasticity remain elusive. In this study, we identify IGF2BP2 as a master m6A-dependent regulator orchestrating this aggressive phenotypic transition. Integrative analysis of RNA-seq, IGF2BP2 RIP-seq, and m^6^A methylome profiling showed that IGF2BP2 post-transcriptionally silencing *STAT1* by binding to m6A-modified sites, thereby accelerating its decay. Notably, we identify STAT1 as a direct transcriptional activator of thyroid differentiation genes (*TSHR*, *SLC26A4*, *SLC5A5*, *TPO*, *PAX8*,* FOXE1*, and *NKX2.1*) through promoter binding, a novel regulatory axis that has not been previously characterized. This IGF2BP2-STAT1 axis critically enforces the loss of stem-like traits and promotes terminal differentiation in thyroid cancer cells.

In the present study, we emphasize the role of IGF2BP2 in facilitating thyroid cancer dedifferentiation and stemness sustaining. The oncogenic role of IGF2BP2 extends beyond THCA, with its overexpression and functional dominance reported in diverse malignancies. In acute myelocytic leukemia (AML), IGF2BP2 maintains the function of human and murine leukemia stem cells (LSCs) via stabilizing *PRMT6* mRNA in m^6^A-mediated manner(37. Similarly, IGF2BP2 promotes lung cancer radio-resistance by forming a positive feedback loop with SLC7A5, enhancing its stability and translation through m^6^A modification^27^. IGF2BP2's pro-tumorigenic role is highly preserved in head and neck squamous cell carcinoma (HNSCC). Yu *et al*. demonstrated that IGF2BP2 recognizes m^6^A modifications within the coding sequence (CDS) of *Slug* mRNA, stabilizing its transcript to drive epithelial-mesenchymal transition (EMT) and metastasis^28^. Extending these findings, Cai *et al*. reported that IGF2BP2 drives HNSCC tumorigenesis and metastasis through super-enhancer (SE) activation (H3K27Ac/BRD4/MED1) and KLF7-mediated transcriptional co-regulation^29^.

The pro-tumorigenic function of IGF2BP2 in thyroid cancer has been well documented in multiple studies. Sa *et al.* reported that IGF2BP2 stabilizes* ERBB2* mRNA in an m6A-dependent manner, thereby conferring radioiodine resistance in PTC 38. Wang *et al.* demonstrated that it promotes lymph-node metastasis by protecting *DPP4* transcripts 39, while Dong *et al.* showed that IGF2BP2 sustains tumor growth via the lncRNA *HAGLR* axis 40. These findings indicate that IGF2BP2 drives both proliferation and metastasis in thyroid cancer. Collectively, these studies establish IGF2BP2 as a key oncogenic driver in thyroid cancer, however, they have examined PTC and ATC as separate entities without considering the continuous differentiation spectrum that characterizes thyroid tumors. Mounting evidence has demonstrated that tumors comprise heterogeneous cell populations with diverse differentiation states including a small portion of CSCs alongside a larger number of deferentially differentiated cancer cells^32^,^33^. Luo *et al.* depicted the heterogeneity of THCA from a single-cell perspective, providing direct evidence that ATC cells originate from a small subset of PTC cells^4^. Expanding on these studies, we proposed that dynamically escalated *IGF2BP2* expressions during dedifferentiation might signify its critical regulatory role in this process. Correlation analysis with TDS and pseudotemporal trajectory mapping further supported the assumption. Diverging from conventional approaches that prioritize downstream effectors through database mining or transcriptomic profiling of static differentiation states^12^,^13^,^16^,^17^, we unpinned 23 genes as conserved downstream targets across differentiation hierarchies by intersecting DEGs from *IGF2BP2*-overexpressing PTC and *IGF2BP2*-knockdown ATC models. Multi-omics integration using RNA-seq, RIP-seq, and m6A methylome profiling has identified STAT1 as the direct m6A-dependent target of IGF2BP2.

The dual role of STAT1 in cancer has long been debated, with context-dependent pro- or anti-tumorigenic effects reported across malignancies^41^-^45^. While STAT1 is widely recognized as a mediator of interferon (IFN)-driven immune responses and tumor surveillance^46^, its cell-intrinsic functions in epithelial differentiation programs remain poorly characterized. In this study, prognostic analysis and functional phenotypic experiments across thyroid carcinoma differentiation subtypes revealed a tumor-suppressive role of STAT1. Mechanistically, we demonstrate that STAT1 directly binds to promoters of thyroid differentiation markers (*TSHR, SLC26A4, SLC5A5, TPO, PAX8, FOXE1, and NKX2.1*) and activates their transcription, thereby restricting stemness and disease progression. This cell-autonomous differentiation-promoting role sharply contrasts with STAT1's reported oncogenic activity in TNBC 47 and nasopharyngeal carcinoma^48^, where it facilitates chromatin accessibility and transcriptional activation of *PDL1* and *IDO1*. Such functional duality may stem from STAT1's differential post-translational modifications (e.g., phosphorylation vs. acetylation) or chromatin accessibility patterns in distinct tissue contexts.

### Limitations of the study

While our study provides critical insights into the IGF2BP2-STAT1 axis in thyroid cancer dedifferentiation and stemness, several limitations warrant acknowledgment. First, although single-cell transcriptomic analysis suggested IGF2BP2's role in driving dynamic dedifferentiation trajectories, experimental validation of its spatiotemporal contributions to this process remains incomplete. The lack of longitudinal models, such as thyroid cancer organoid systems or time-resolved *in vivo* experiments, limits our ability to mechanistically dissect how IGF2BP2 orchestrates dedifferentiation during tumor evolution phases. Second, while cancer stem-like properties are widely recognized to confer resistance to conventional therapies through mechanisms such as enhanced drug efflux or dormancy, our study did not explore the functional relationship between IGF2BP2-driven stemness and therapeutic outcomes in preclinical models. Third, the genetic backgrounds of the cell models used in this study should be considered. While our functional experiments demonstrated that the pro-dedifferentiation effects of the IGF2BP2-STAT1 axis are consistent across both BRAF wild-type and BRAF V600E mutant PTC models, all ATC models utilized were BRAF wild-type.

Future investigations should incorporate time-resolved studies in genetically engineered animal models or patient-derived systems to 1 dynamically delineate the spatiotemporal role of the IGF2BP2-STAT1 axis in dedifferentiation trajectories , 2 determine whether therapeutic inhibition of this pathway restores differentiation signatures and reverses radioiodine-refractory or drug-tolerant phenotypes and 3 employ BRAF-mutant ATC models, such as patient-derived organoids or rare cell lines, to conclusively determine if this mechanism operates independently of the BRAF oncogenic driver in the most advanced disease state.

## Conclusion

In conclusion, our study elucidates a novel thyroid-specific regulatory axis involving IGF2BP2 and STAT1 that drives dedifferentiation and enhances stemness in diverse subtypes of differentiated thyroid cancers. These findings position the IGF2BP2-STAT1 pathway as a central molecular mechanism underlying tumor progression and loss of differentiation, which are critical determinants of therapeutic resistance and poor prognosis in THCA. Therapeutic interventions targeting this axis may be promising to restore differentiation, suppress stem-like properties, and potentially reverse treatment refractoriness in advanced or radioiodine-resistant thyroid malignancies.

## Supplementary Material

Supplementary figures and tables.
