# Supplementary material for: IGF2BP2 Drives Thyroid Cancer Dedifferentiation Through m6A-Dependent STAT1 mRNA Destabilization
Source: Int J Biol Sci. 2026 Jan 1;22(2):622–40. doi: 10.7150/ijbs.121503 (PMC12780945; doi:10.7150/ijbs.121503)
Supplement: Supplementary file 1 — Supplementary figures and tables. [file ijbsv22p0622s1.pdf]

Supporting Information

IGF2BP2 Drives Thyroid Cancer Dedifferentiation Through m6A-Dependent STAT1 mRNA Destabilization

Rui Chen[1]†, Yi-xun Li[1]†, Wei-lin Lu[1]†, Ke-fei Wu[1], Yu-xin Wang[1]， Zi-wen Wang[1], Yi-han Li[1], Hai-yan Yang[1]， Xu Zhang[1]， Liang Shi[1], Dong Zhou[2,3], Ying Wang[4]*, Qiang Ding[1]**

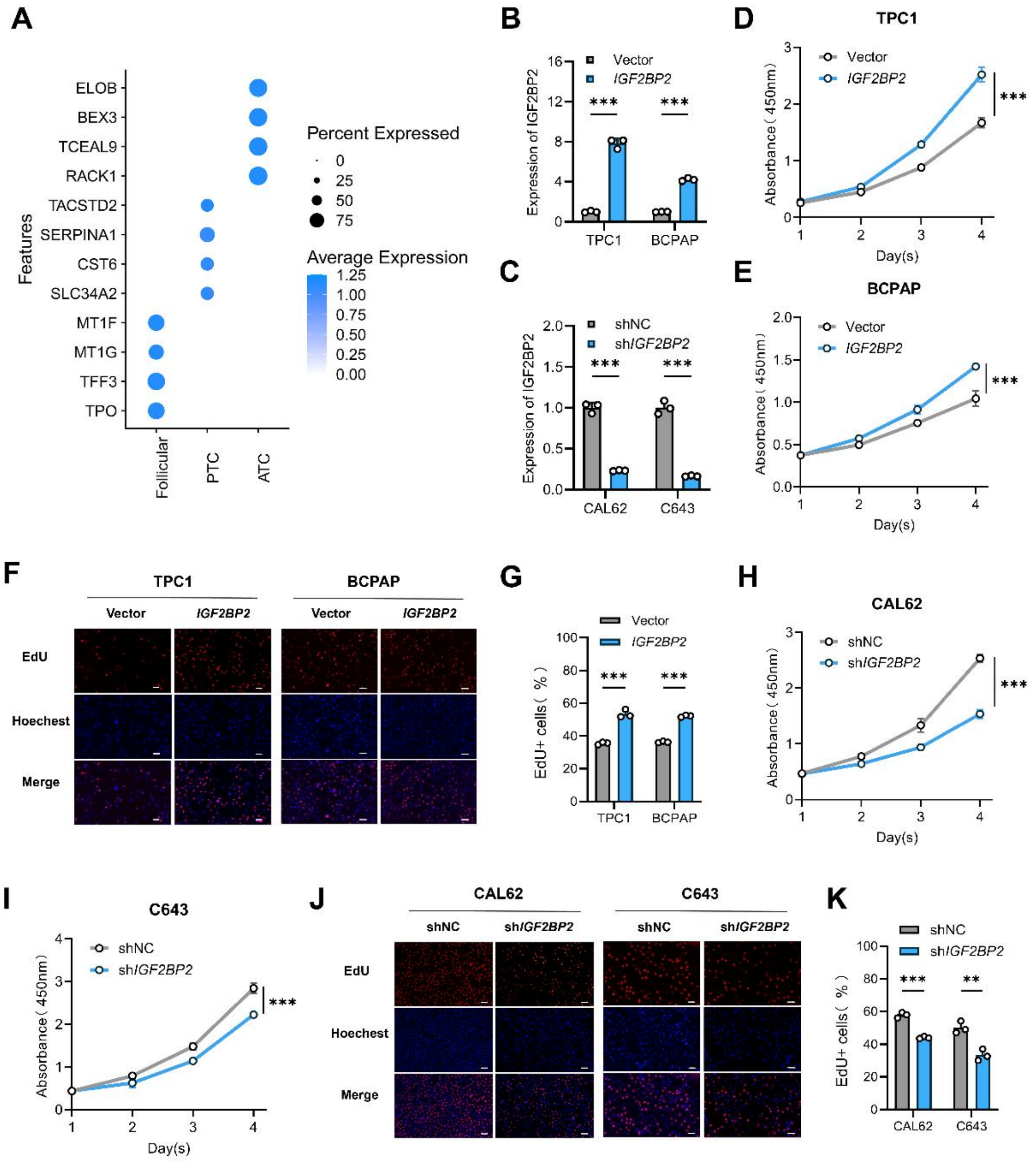

Fig. S1. (Related to Figure 2): IGF2BP2 promoted thyroid cancer proliferation

(A) The bubble plot illustrates genes specifically enriched in normal follicular, PTC, and ATC clusters. (B-C) The efficiency of stable *IGF2BP2* overexpression and knockdown cell lines. (D-G) CCK8 and EdU assays performed in TPC1-OE and BCPAP-OE cells. (H-K) CCK8 and EdU assays performed in CAL62-KD and C643-KD cells. *P* values were determined using a two-tailed unpaired Student's test (* $P < 0.05$, ** $P < 0.01$, *** $P < 0.001$).

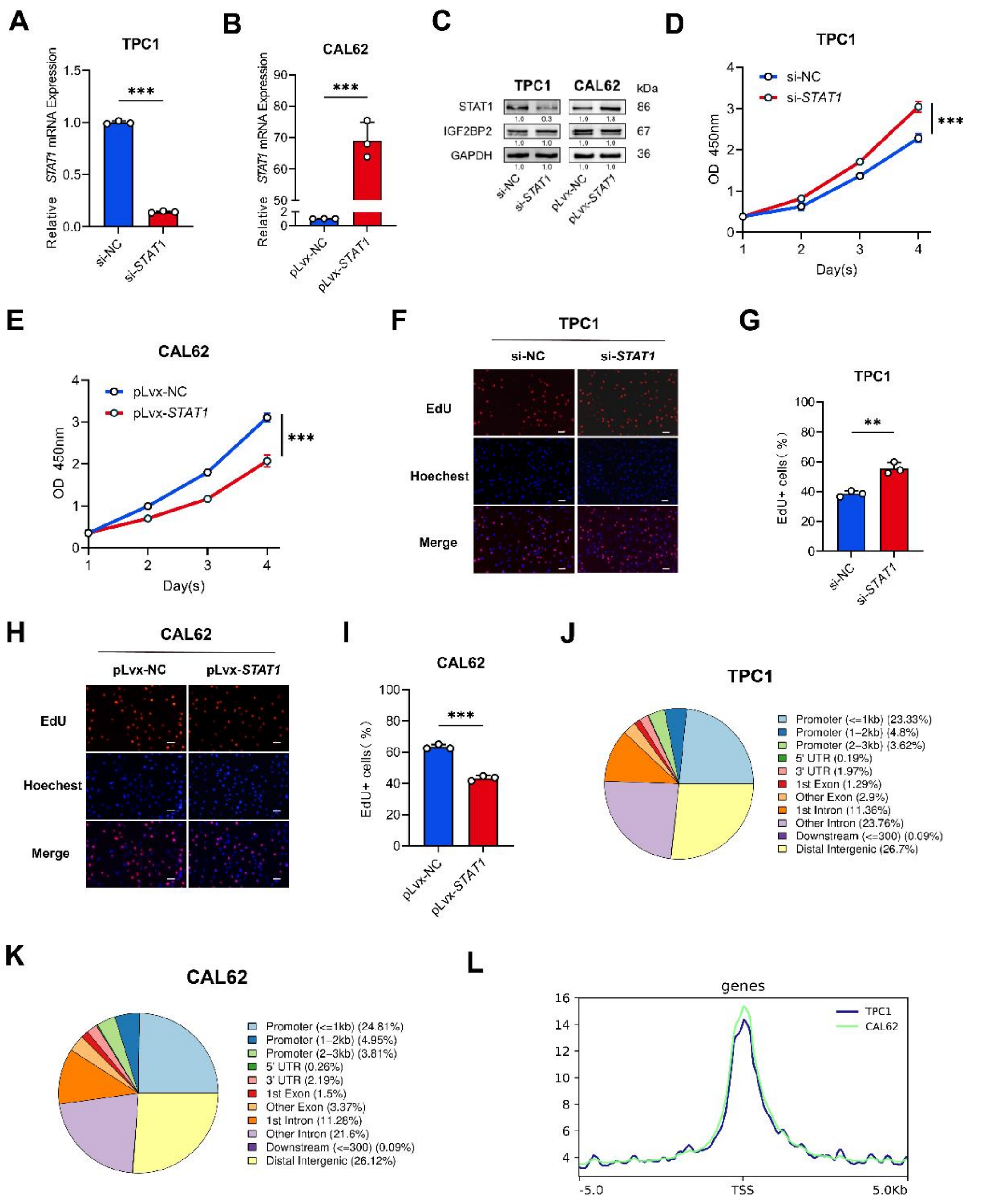

Fig. S2. (Related to Figure 6): STAT1 impaired the cell growth of thyroid cancer

(A-C) Wildtype TPC1 and CAL62 cells were transfected with *STAT1* targeted siRNAs and overexpression plasmids for 48 h. Transfection efficiency was verified by qRT-PCR (A-B) and western blot (C). (D) CCK8 assay using TPC1 transduced with *STAT1* siRNAs. (E) CCK8 assay using CAL62 transduced with *STAT1* overexpression plasmids. (F-G) EdU assays using TPC1 transduced with *STAT1* siRNAs. (H-I) EdU assays using CAL62

transduced with *STAT1* overexpression plasmids. (J-K) Pie chart of genome-wide distribution of upregulated STAT1 enrichment peaks in TPC1 and CAL62 cells. (L) The distribution density of STAT1-binding peaks around TSS in TPC1 and CAL62 cells. *P* values were determined using a two-tailed unpaired Student's test (* $P < 0.05$, ** $P < 0.01$, *** $P < 0.001$).

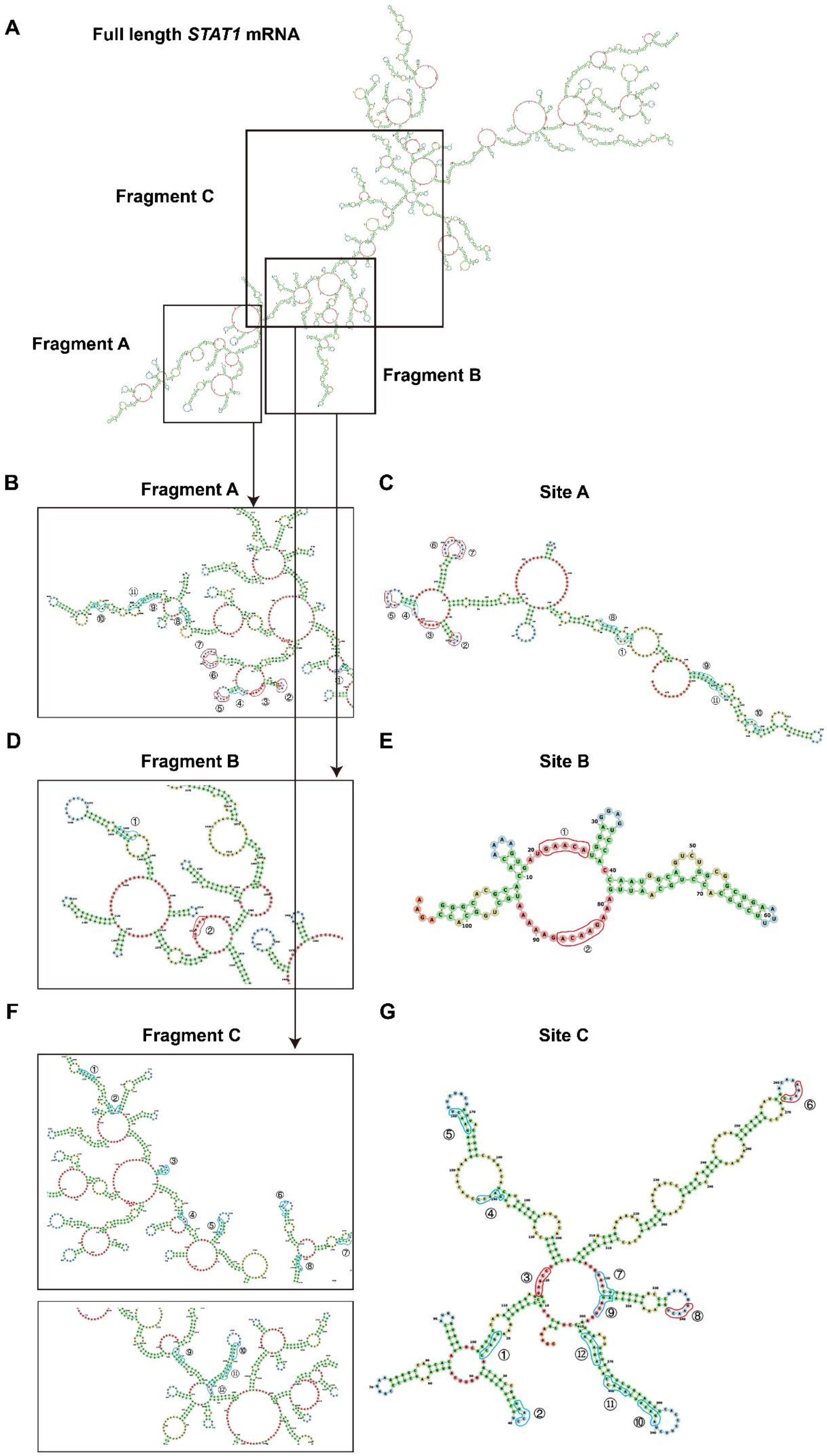
A
Full length STAT1 mRNA
Fragment C
Fragment A
Fragment B
B
Fragment A
C
Site A
D
Fragment B
E
Site B
F
Fragment C
G
Site C

Fig. S3. (Related to Figure 7): Comparative *in silico* analysis of secondary structures for the *STAT1* m6A sites

(A) Secondary structures of the full-length *STAT1* mRNA were predicted using RNAfold (http://rna.tbi.univie.ac.at//cgi-bin/RNAWebSuite/RNAfold.cgi). (B) The local RNA secondary structures of the high-confidence m6A site A within the full-length *STAT1* mRNA was predicted using RNAfold. (C) The local RNA secondary structures of the isolated site A was predicted using RNAfold. (D) The local RNA secondary structures of the high-confidence m6A site B within the full-length *STAT1* mRNA was predicted using RNAfold. (E) The local RNA secondary structures of the isolated site B was predicted using RNAfold. (F) The local RNA secondary structures of the high-confidence m6A site C within the full-length *STAT1* mRNA was predicted using RNAfold. (G) The local RNA secondary structures of the isolated site C was predicted using RNAfold. Accessible RRACH motifs are highlighted in red, while inaccessible motifs are colored blue.

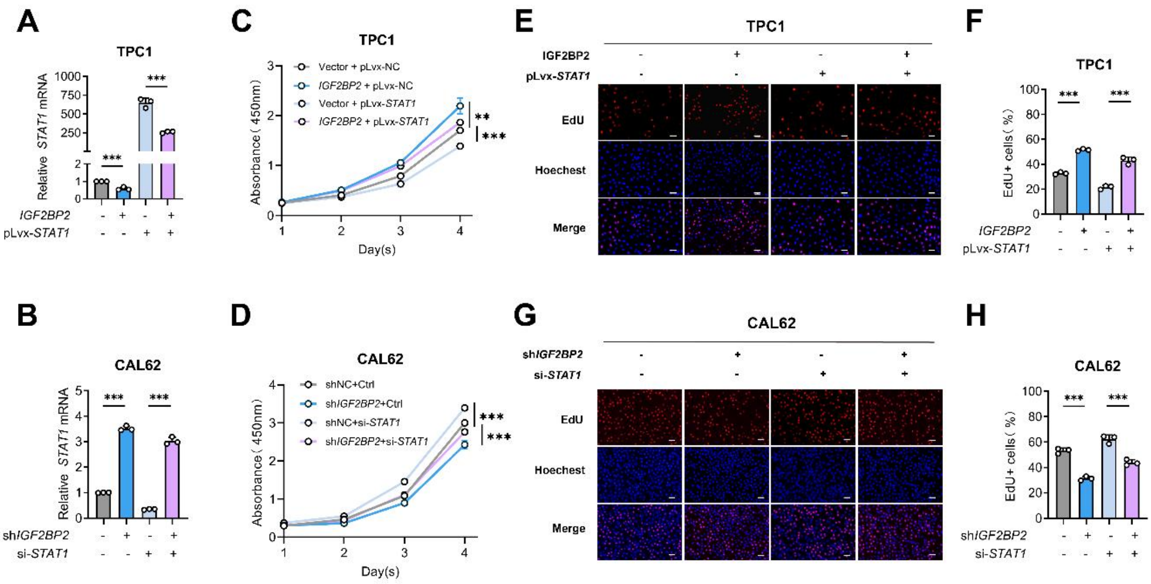

Fig. S4. (Related to Figure 8): STAT1 reversed the vicious proliferation promoted by IGF2BP2 in thyroid cancer

(A-B) The TPC1-OE cells were transfected with pLvx-*STAT1* plasmids and CAL62-KD cells were interfered with si-*STAT1*, confirmed by qRT-PCR. (C) CCK8 assays showing the rescue effect of *STAT1* overexpression on TPC1-OE cells. (D) CCK8 assays showing the rescue effect of *STAT1* interference on CAL62-KD cells. (E-F) EdU assays showing the rescue effect of *STAT1* overexpression on TPC1-OE cells. (G-H) EdU assays showing the rescue effect of *STAT1* interference on CAL62-KD cells. *P* values were determined using a two - tailed unpaired Student's test (* $P < 0.05$, ** $P < 0.01$, *** $P < 0.001$).

Table S1.

The sequence information of Lentivirus, and siRNA. Related to Methods.

**shRNA sequences (5'-3')**	
shIGF2BP2	ACAGGACUGUCCGUGCUAUTT
**siRNA (5'-3')**	
si-STAT1	CCCTGAAGTATCTGTATCCAA
si-CNOT1-1	TGCCTATTTGGTGGTATAATT
si-CNOT1-2	CATTCAACATTCCCTTATAAA

Table S2.

The sequence information of qPCR primers. Related to Methods.

**qPCR primer sequence (5'-3')**		
ACTIN	Forward	CATGTACGTTGCTATCCAGGC
	Reverse	CTCCTTAATGTCACGCACGAT
IGF2BP2	Forward	AGTGGAATTGCATGGGAAAATCA
	Reverse	CAACGGCGGTTTCTGTGTC
STAT1	Forward	CAGCTTGACTCAAAATTCCTGGA
	Reverse	TGAAGATTACGCTTGCTTTTCCT
TSHR	Forward	GGAATGGGGTGTTCGTCTCC
	Reverse	GCGTTGAATATCCTTGCAGGT
TPO	Forward	GCCAACAAGCGGAGTGATTG
	Reverse	GGGCAGCATGTAAGGGAGAC
FOXE1	Forward	CACGGTGGACTTCTACGGG
	Reverse	GGACACGAACCGATCTATCCC
PAX8	Forward	ATCCGGCCTGGAGTGATAGG
	Reverse	TGGCGTTTGTAGTCCCCAATC
NKX2.1	Forward	AGCACACGACTCCGTTCTC
	Reverse	GCCCACTTTCTTGTAGCTTTCC
SLC5A5	Forward	GCAGTACATTGTAGCCACGAT
	Reverse	TGCAGATAATTCCGGTGGACA
SLC26A4	Forward	GCTCCCCAAATACCGAGTCAA
	Reverse	CACCACTGGAAAAGGTCCAAC
CD133	Forward	AGTCGGAAACTGGCAGATAGC
	Reverse	GGTAGTGTTGTACTGGGCCAAT
SOX2	Forward	TGGACAGTTACGCGCACAT
	Reverse	CGAGTAGGACATGCTGTAGGT
OCT4	Forward	CTGGGTTGATCCTCGGACCT
	Reverse	CCATCGGAGTTGCTCTCCA
NANOG	Forward	AAGGTCCCGGTCAAGAAACAG
	Reverse	CTTCTGCGTCACACCATTGC
CNOT1	Forward	ACTTCCTTGACACGCTGAAGA
	Reverse	ATTGGCTGCAGCTTTATCAGA
**Methylation-specific primers (5'-3')**		
STAT1	Forward	GCCCAGAGATTTATGGTCTCGT
	Reverse	CAGGCTCTTGATTTCATGCTCT

Table S3.

The sequence information of Luciferase. Related to Methods.

**Plasmids: Luciferase sequence (5’-3’) in Fig. 6**	
STAT1	GCCACCATGTCTCAGTGGTACGAACTTCAGCAGCTTG<br>ACTCAAAATTCCTGGAGCAGGTTCACCAGCTTTATGA<br>TGACAGTTTTCCCATGGAAATCAGACAGTACCTGGCA<br>CAGTGGTTAGAAAAGCAAGACTGGGAGCACGCTGCC<br>AATGATGTTTCATTTGCCACCATCCGTTTTCATGACCT<br>CCTGTCACAGCTGGATGATCAATATAGTCGCTTTTCTT<br>TGGAGAATAACTTCTTGCTACAGCATAACATAAGGAA<br>AAGCAAGCGTAATCTTCAGGATAATTTTCAGGAAGAC<br>CCAATCCAGATGTCTATGATCATTTACAGCTGTCTGA<br>AGGAAGAAAGGAAAATTCTGGAAAACGCCCAGAGAT<br>TTAATCAGGCTCAGTCGGGGAATATTCAGAGCACAGT<br>GATGTTAGACAAACAGAAAGAGCTTGACAGTAAAGT<br>CAGAAATGTGAAGGACAAGGTTATGTGTATAGAGCA<br>TGAAATCAAGAGCCTGGAAGATTTACAAGATGAATA<br>TGACTTCAAATGCAAAACCTTGCAGAACAGAGAACA<br>CGAGACCAATGGTGTGGCAAAGAGTGATCAGAAACA<br>AGAACAGCTGTTACTCAAGAAGATGTATTTAATGCTT<br>GACAATAAGAGAAAGGAAGTAGTTCACAAAATAATA<br>GAGTTGCTGAATGTCACTGAACTTACCCAGAATGCCC<br>TGATTAATGATGAACTAGTGGAGTGGAAGCGGAGAC<br>AGCAGAGCGCCTGTATTGGGGGGCCGCCCAATGCTTG<br>CTTGGATCAGCTGCAGAACTGGTTCACTATAGTTGCG

GAGAGTCTGCAGCAAGTTCGGCAGCAGCTTAAAAAG

TTGGAGGAATTGGAACAGAAATACACCTACGAACAT

GACCCTATCACAAAAAACAAACAAGTGTTATGGGAC

CGCACCTTCAGTCTTTTCCAGCAGCTCATTCAGAGCT

CGTTTGTGGTGGAAAGACAGCCCTGCATGCCAACGCA

CCCTCAGAGGCCGCTGGTCTTGAAGACAGGGGTCCA

GTTCACTGTGAAGTTGAGACTGTTGGTGAAATTGCAA

GAGCTGAATTATAATTTGAAAGTCAAAGTCTTATTTG

ATAAAGATGTGAATGAGAGAAATACAGTAAAAGGAT

TTAGGAAGTTCAACATTTTGGGCACGCACACAAAAGT

GATGAACATGGAGGAGTCCACCAATGGCAGTCTGGC

GGCTGAATTTCGGCACCTGCAATTGAAAGAACAGAA

AAATGCTGGCACCAGAACGAATGAGGGTCCTCTCATC

GTTACTGAAGAGCTTCACTCCCTTAGTTTTGAAACCC

AATTGTGCCAGCCTGGTTTGGTAATTGACCTCGAGAC

GACCTCTCTGCCCGTTGTGGTGATCTCCAACGTCAGC

CAGCTCCCGAGCGGTTGGGCCTCCATCCTTTGGTACA

ACATGCTGGTGGCGGAACCCAGGAATCTGTCCTTCTT

CCTGACTCCACCATGTGCACGATGGGCTCAGCTTTCA

GAAGTGCTGAGTTGGCAGTTTTCTTCTGTCACCAAAA

GAGGTCTCAATGTGGACCAGCTGAACATGTTGGGAG

AGAAGCTTCTTGGTCCTAACGCCAGCCCCGATGGTCT

CATTCCGTGGACGAGGTTTTGTAAGGAAAATATAAAT

GATAAAAATTTTCCCTTCTGGCTTTGGATTGAAAGCA

TCCTAGAACTCATTAAAAAACACCTGCTCCCTCTCTG

	GAATGATGGGTGCATCATGGGCTTCATCAGCAAGGA<br>GCGAGAGCGTGCCCTGTTGAAGGACCAGCAGCCGGG<br>GACCTTCCTGCTGCGGTTCAGTGAGAGCTCCCGGGAA<br>GGGGCCATCACATTCACATGGGTGGAGCGGTCCCAG<br>AACGGAGGCGAACCTGACTTCCATGCGGTTGAACCCT<br>ACACGAAGAAAGAACTTTCTGCTGTTACTTTCCCTGA<br>CATCATTCGCAATTACAAAGTCATGGCTGCTGAGAAT<br>ATTCCTGAGAATCCCCTGAAGTATCTGTATCCAAATA<br>TTGACAAAGACCATGCCTTTGGAAAGTATTACTCCAG<br>GCCAAAGGAAGCACCAGAGCCAATGGAACTTGATGG<br>CCCTAAAGGAACTGGATATATCAAGACTGAGTTGATT<br>TCTGTGTCTGAAGTTCACCCTTCTAGACTTCAGACCA<br>CAGACAACCTGCTCCCCATGTCTCCTGAGGAGTTTGA<br>CGAGGTGTCTCGGATAGTGGGCTCTGTAGAATTCGAC<br>AGTATGATGAACACAGTATAG
TSHR	CTGCAACGTGAGGCGGCCGCTGCCAGTCGACTCAACC<br>ACCGGAGTGGCCCCTGCAGTTGGATAGCAACGAGAA<br>TCCTCCAGGGGTGCAGGGCGACGGCTTCGGCCGCACC<br>GCGGGCTAGCCAGGGCTGCGTGCCCGCCTCTGACCCT<br>CAGCAGAGGTGTCTCTGGCCAGGAGGAGCTGAAGTT<br>CTGCAGGACATTGGTCCGCCCGCGGACAGTCCACTCC<br>GCGGGGACTTTCTCTGGATAAGGAGTGCGTGCGAGTG<br>GCTCCCAGGCAGACAGGGTGTCTAGAAGGCTACACG<br>CTAGGGAAGGTGGCTCCTTGGATTTAAAGAGGAGGA<br>AAGGAGGGGGCATCTAAACTAGGCTTTGGAGAGAAC

	TAATGGGAGGGGCGCCCGGGGTGGGGGGCGGGCTG<br>GAAAACAGAGGGGACAGCCAGGACTGGTGTTGGG
SLC26A4	GTACAGAGGTCTGTATTTTGGAGCCTCTTCTGTATTTA<br>CTTCAGAACACTAACAATCAGGCGAGAATGTTCTGGT<br>TTATCAAACCCTTCCTTCTGCCTTTCATCTTAACCATG<br>CATTAGTTTTAACAAAGTTCATCCCAACAGAAGACAA<br>AACACTGATGAGGTAGGATAGCTCCAGCTCCTCCTCC<br>CTCTCTTCTAGTCTTGATTTCCATGTAGTCCAGTTTAT<br>TCCTTCCCTGATTGTCCAGGAGAATGAGAAAAAGAA<br>AAAACAGAGTCTAGTGGGTAAGAAAGGGCCACCTGG<br>ACGGCTTGATTTGGATTGTGAAATAAAACACACACAC<br>ATGCACACGTAGAATAAGTGGCTAAAATCTGAGTAA<br>ATCGTGAACTCTCTGTATCCTCCACCCATTGAATACTC<br>CTAAAAGACTTTCTAGAAATTCAAGGACTTATTAATA<br>TAGAAACCTGGCCATTGTTCCTCTTCTCCTCCCCATGT<br>GGTATGAGAGCACCTGTGGCAGGCTCCCAGAGACCA<br>CGGACCTCTTCCTCTAGGCGGGCTCTGCTCTTCTTTAA<br>GGAGTCCCACAGGGCCTGGCCCGCCCCTGACCTCGCA<br>ACCCTTGAGATTAGTAACGGGATGAGTGAGGATCCG<br>GGTGGCCCCTGCGTGGCAGCCAGTAAGAGTCTCAGCC<br>TTCCCGGTTCGGGAAAGGGGAAGAATGCAGGAGGGG<br>TAGGATTTCTTTCCTGATAGGATCGGTTGGGAAAGAC<br>CGCAGCCTGTGTGTGTCTTTCCCTTCGACCAAGGTGT<br>CTGTTGCTCCGTAAATAAAACGTCCCACTGCCTTCTG<br>AGAGCGCTATAAAGGCAGCGGAAGGGTAGTCCGCGG

	GGCATTCCGGGCGGGGCGCGAGCAGAGACAGGTGAG<br>TTC
SLC5A<br>5	GGACTGTACTCTCTGCCTCACAGTCTTCATCAATAAA<br>ATGAGGATGACACGGGTCCCTGCTTCACAGGGATCCC<br>AGGGCTCAGAACACAGTGGGGCTCAGGGACAATTCT<br>CAGGTTCATGCAGTTGATCAACAAATGCACATTATAG<br>CCTGAGGAACATAGGGAGACCCCATCTTTACACAATT<br>TTTTTTTTTTGAGCCAGAGTCTCACTCTGTTGCCCAG<br>GCTGGAGTGCAGTGGCGCAACCTCAGCTC
TPO	ACCACCCCAGCTGGCCTTCGAGGCCTCACGGGTCTCC<br>GGCACTGGGTCGCCTGAGCACTGCCCAGGTTCGCACA<br>TTCTCATCGTCCTGGGGTGGCCGAGTCGGGGACCAAG<br>ACCCTGTGGCCTATGTGGACCCCGGCTCCTCCACTCA<br>GCGGTGAGACTGGGGAGGGTCCCTCCACCTCTCCCAG<br>TGCTTTTGCTCTGTCTAGGGATGTCGTCAGCACCAGG<br>GCCCCAGGAGTGCAGGGAGGTTGCTGCCTCCAGCCTG<br>GCCCTCGGCGGAGGCTGCACCCAGGCCAGCTCTGTTC<br>CCTCCAGACAGAGCAGCTCCTGGAAGAGCTCGTGTTC<br>CCCACACTGACAGCACCTGCTCCATTTAGAGAATGCG<br>CACTGGGCATGTAGAGAAGACCTGTGACACGCTACA<br>GCTTCTTTTCTAAAACTGGCCAGAAAAGGAACATTAT<br>TTGAACAAAATGTACAGCAGGTTATCTGGAAACATAC<br>GCCTAGGGGATGAGATACTAGGTTGTAGGATTCTTGT<br>GCAGGGGGAGCAGGAGCAGGAGGGGACCCCACATCC<br>TCACTGGCGGTGGCAGGTGGCCCCAGTCCTTGCTCTC

	AGAGGACCTGCCGGCCAGCTGGGAGCATCTGCCTGG<br>GCGAAGCAGACCCTGCCCCACCCTCACCC
PAX8	GTGCTGGGACAGGAGGGCAGCGGGCGGTGCACGAGA<br>GCAGTTTGCTCAGGCTTTGATGGGGACAGAAACCCTT<br>CCACTTATGATATGGGACTTTGTAGGGGCTCTGCATG<br>TGACTGTCACATGTCCTGTCCCTCAGTTGGCCAAGGC<br>TCCTGGGCCCACGTGGCTGGCCCTTTTTCTCCCTCCAC<br>AGTGAGTCAGCTTGGAGTCAGTCCTCTACTTTGGCCT<br>AGAGCATGAATAGATAGGTCCCCTAAAAGCTTTCTTC<br>CTTCCCGGGGGGTACCAGGTGATCAGGATAGCTGCCG<br>ACTAAGCATTGACTCACAGAGCAGCAGCAGCGGACC<br>CCGAAAGCACCTTCGCACGGATGCCTTCAGCCAGCAC<br>CACCTCGAGCCGCTCGAGTGCCCATTTGAGCGGCAGC<br>ACTACCCAGAGGCCTATGCCTCCCCCAGCCACACCAA<br>AGGCGAGCAGGTGAGAAGCTGGGCCCTGGGAGGTGA<br>ACAGGGTGGGCAAGGGCCAGAGAAGGTCTATTCTCC<br>AAATGAGCCTGTGCTCCATCAAAGCAGCTGGAAGTTG<br>CATCAATGGGCTCAGGGCACCTGCCTTATGCAACTCC<br>AAGCCTGACACATGCTTTATTTACCAGATGCCCTGG<br>CATCTTCCTCCCTTTCTAACCCTCCTGGCTTCCTTCAC<br>CCAAAACCTTTGAGCCTGGGCCTTTCAGGGATGGCAG<br>CACAACCTGATGATGCTATCCCTTGCCATCAGCGTCT<br>TTCCCAGATTGCAACATCACGTTCCACAACTCTGAAG<br>CAGTAGCACAAAGCCTTCACATAATGACCTCAGCCAC<br>TATTTCCTAGCCCCACCTTTGGCCACTCATCCTGCACA

	CCCCATGCTCTTGCATTACTCATGGCCCCGAGCTCAC<br>CACCCTCGCTCACACCTCTCTGCCCTTGCACACTGTGT<br>GCCCTCCACACAGAATGCCTTTCCTCCCACTTCTCTGC<br>ATGGAAAAATACCACTAGCCTTCATGACTCAGCCAAA<br>CATCACCCTCTCCGTCAGTCCTCCCCTGCCCCTCCGAC<br>ATGAATCATCTCTCCTGCCTCTTGCCCTTCTGCATGTC<br>CCACATGCTGCTCTCAGTAGCTTCTCACAGTGTCACTT<br>AATAATCTAGCAGACAGACAGGTACAAAGACACGCG<br>GACTCCTTAAGGACAGGGAAGCCTCATGAAACGTTA<br>AACATCGTTTTTATTCCCAGCACCTACCATTGTGCCCC<br>CCTTATAATAGACAGTAATGGTATAGTGAATGAATGA<br>ACAAATGAATGAGTTGAGAAATCCAGAATAATACAT<br>CACAAGCTACAGCGAATTTTTCTTGCTAGTGCTCAGG<br>ACAGGTGAAATGAATCATAACAGGTTGGTGTCCGTG<br>AAAAGCTGACAGAGATGTGAGA
NKX2.1	GATGAGAGGCGCCCAATTGAAGCAGAATGATCCTCA<br>TCTACTAATATCCAGCGTGGCCACAAAGCGACTGGCC<br>ATTTACGCCACCACTTTAAACAAAGATATTTGGTTAT<br>TCCCGGGAAGCAAGAGCACTTTTGCATGGCTGAGCTG<br>CGGGCGGAGGCGAGCCTCGGCCCAGCCTCCCGCCCG<br>CTGAGCTGCCCGCACCTCGGCATTCGCCCCCTCCTGG<br>CCAGCGCTCCAGCCGCCGGGGTTCAGGCTCAGTTCCG<br>CCCGGCAACAGACAGACGGGCACTCACGCCGCTCCC<br>TGCTCGCGCGCTCCCTGAAGCATCAGAGGGGAAAAC<br>AGCGTGGCTCTGGGCTCGGGTGCTGGGGCTGTGATGT

	CCTCGGAAAGTCAGCTCCAGCCCCAGATC
FOXE1	AGGCTCCCGCCACCGTGCCTGGTTTAGTCCCAGTATC<br>TTAATACATAGGTTTTTAGACTTTGACTAGAAGCTTA<br>AGTCAAAGGAATATAAGCATAGTCAAAGTTAGACTC<br>CCAGTCTTGGAATCTTAGAATTACAGGGTCGCAGGAT<br>CGCAGGATCGCATTCTTTAGATTTATGGAATCACGTT<br>AGGACAACGGTCGTCCAGAATTTAAAATCTAGATATC<br>TGAATCTCAGAATCTTGAAAGTTACTGAGAGCCACGA<br>AATCCCGTATTAAAAGGTGCCTATAATATAATCCAGT<br>TAAAAATTTTAAAATTTGAAGTTTTTCCTCCTTTGTAA<br>AAACGAAACAGGACGAAATCTGGGTTTTTTGCTTCTC<br>CATTACTTGGGAAGAAGGAAACCAGGAGTTTTGTTTA<br>GCGAGTGTAAAGCCCCTCTCCCTTCGTTCGACCAACC<br>CTCTGCCGCAGAAGGTTGTCAAGCAAACGCTTTGAGC<br>GTTTCCACACACCGGGTCGACGGAGCAAGGATCTGG<br>GCTTTGCCTCGCTCTTCCGAAGGCAGCTGCCCAACGC<br>TTGGCCGGGCTTAGCCTGCCCTCAGCTCACCGCCACG<br>AAGAACGCGACTAAAACCCTGGAAGGCATGCCACCC<br>GTTACTGGCCAGAACTCTCGTCTTGGGATCTCCCACA<br>CTCACCTGGCACCACCATCCGCGCGGCTCTGCACCCA<br>CAGCCCCGGCCCGCCCCCCCCCCCACCCCGGAAAGC<br>TGCGTCCGGGCTGGAGCCACTGGAACCCGCGCCAAG<br>GCCCGGAATCCTATACGTAGCAGGGGCCTCGGAGAT<br>CAGCACACGCCCTCCAGCTGCTATTTAACAGAGTAGA<br>ACACTGAGGCCCTGCGAGGGGACAAGGACAGGCCCT

GGATCTCCCAGTGAATGGCCAGGGAACGAACCCGGG

CCAGAGGGGCCGCGCCGCGAGGATCTCAGGTTAGGA

CCAAGTTCCGGCTCAGGGACAGCAGGAAAGGAACTC

AGAAATTGGACACCCATGAAGCAAACGTGTCCCGAC

TGCCCGCCCCTTCCCCCGGAGACGCGCCCACCCGGCC

ACCGCTCTCTTCCCACTCCCCCATTACCCGCAGCCCTC

ACTCCCCGCTGCGGGAAGGGGCTGCTTGGCTGCCTCT

GGGGGTCTTCAGAGCTACCCTGGTCCCGGGGGATTGG

AGGAGGAGGTTACCTATCCTGCGTCGTCTTTAATCCG

TGCACCACTATCCATCAAATAGAGACAGATCCTGGGC

CTCTCAAAGACGGATGATTGGGGGTGGTGATTGGCCT

ATCCCTAAATATCTACCACGCAAGGACTCTTGAGAGA

TCCAGACCCCGGTACAGTCGAGGGACCTGGGGCCCA

AAAAGGGAAAGCGGCTACCTCTACCACACAGTTGGG

AAGCGCAGTCCTAAAGGAGACGCAGGTTGGAGACTC

CGCTAAGCGGAGAAGCCGCAGTGGGGCCATGGCAAG

TCACCTTCCCTTTCGGGCCTAGGAATACTCATTCGAA

AGATGGGGGGACTGGAGTGCCGAGTGGCTGTGGCAG

CCACGATTGGGGTTTGGAAACCATCCTGAAAGGCCCG

GGGAGCCAGTCTCCTGGAACTTCTCCCTCCCCATTCC

CACAAAAACCAAGCGCCCTCTCGGCCAATTCTCACCC

TCTCAGGACAAAAAAGTGAGATGAGCCCGTCCTTTCA

CCTGCGAGTCCAAGCCCTTGGCAGAGGCCTGAAAAG

TCCGAAAACTCCGAGTTCGGGCGCTGAGGTCTCCCGA

GCCGGTTCCTGAACTCTCCGGGCCTCAGTCGATCGGG

GTGCGGAGGGGGCCGACCCGGGGGATCTCCAAGCGC
CCTCCCCGCCCTGACGCTGTGGGGCTCCTACCGCGCC
GCCACAGCTGCTCCTACCTGGGGAGGTGCGCCCGGGC
CCCGGGGGGCGGGCAGTCGGGGGGCGGGCAGGGAAC
CGGTGCCGCCCCACGCTTCGTGGCCCCTTTAAGGAGG
GGAAGCCGGCGGAGGGAG

**Plasmids: Luciferase sequence (5’-3’) in Fig. 7**	
A	AGTAAAGTCAGAAATGTGAAGGACAAGGTTATGTGT<br>ATAGAGCATGAAATCAAGAGCCTGGAAGATTTACAA<br>GATGAATATGACTTCAAATGCAAAACCTTGCAGAAC<br>AGAGAACACGAGACCAATGGTGTGGCAAAGAGTGAT<br>CAGAAACAAGAACAGCTGTTACTCAAGAAGATGTAT<br>TTAATGCTTGACAATAAGAGAAAGGAAGTAGTTCAC<br>AAAATAATAGAGTTGCTGAATGTCACTGAACTTACCC<br>AGAATGCCCTGATTAATGATGAACTAGTGGAGTGGA<br>AGCGGAGACAGCAGAGCGCCTGTATTGGGGGGCCGC<br>CCAATGCTTGCTTGGATCAGCTGCAGAACTGGTTCAC<br>TATAGTTGCGGAG
B	GGGCACGCACACAAAAGTGATGAACATGGAGGAGTC<br>CACCAATGGCAGTCTGGCGGCTGAATTTCGGCACCTG<br>CAATTGAAAGAACAGAAAAATGCTGGCACCAGAA
C	CGAGAGCGTGCCCTGTTGAAGGACCAGCAGCCGGGG<br>ACCTTCCTGCTGCGGTTCAGTGAGAGCTCCCGGGAAG<br>GGGCCATCACATTCACATGGGTGGAGCGGTCCCAGA

	ACGGAGGCGAACCTGACTTCCATGCGGTTGAACCCTA<br>CACGAAGAAAGAACTTTCTGCTGTTACTTTCCCTGAC<br>ATCATTCGCAATTACAAAGTCATGGCTGCTGAGAATA<br>TTCCTGAGAATCCCCTGAAGTATCTGTATCCAAATAT<br>TGACAAAGACCATGCCTTTGGAAAGTATTACTCCAGG<br>CCAAAGGAAGCACCAGAGCCAATGGAACTTGATGGC<br>CCTAAAGGAACTGGATATATCAAGACTGAGTTGATTT<br>CTGTGTCTGAAGTTCACCCTTCTAGACTTCAGACCAC<br>AGACAACCTGC
A-mut	CGTCCCGTCCGCCCTGTGCCGGCCCCGGTTCTGTGTCT<br>CGCGCCTGAAATCAAGAGCCTGGAAGATTTACAAGA<br>TGAATATGACTTCAAATGCAAAACCTTGCAGAACAG<br>AGAACACGAGACCAATGGTGTGGCAAAGAGTGATCA<br>GAAACAAGAACAGCTGTTACTCAAGAAGATGTATTT<br>AATGCTTGACAATAAGAGAAAGGAAGTAGTTCACAA<br>AATAATCGCGTTGCTGCCTGTCCCTGCCCTTCCCCCGC<br>CTGCCCTGCTTCCTGCTGCCCTCGTGGCGTGGCCGCG<br>GCGCCAGCAGAGCGCCTGTATTGGGGGGCCGCCCAA<br>TGCTTGCTTGGCTCCGCTGCCGCCCTGGTTCCCTCTCG<br>TTGCGGCG
B-mut	GGGCCCGCCCCCCCCGTGCTGCCCCTGGCGGCGTCC<br>CCCCCTGGCAGTCTGGCGGCTGAATTTCGGCCCCTGC<br>CCTTGCCCGCCCCGCCCCCTGCTGGCCCCCGCC
